# MicroProtein-Mediated Recruitment of CONSTANS into a TOPLESS Trimeric Complex Represses Flowering in Arabidopsis

**DOI:** 10.1371/journal.pgen.1005959

**Published:** 2016-03-25

**Authors:** Moritz Graeff, Daniel Straub, Tenai Eguen, Ulla Dolde, Vandasue Rodrigues, Ronny Brandt, Stephan Wenkel

**Affiliations:** 1 Center for Plant Molecular Biology, University of Tübingen, Tübingen, Germany; 2 Copenhagen Plant Science Centre, University of Copenhagen, Copenhagen, Denmark; 3 Department for Plant and Environmental Sciences, University of Copenhagen, Copenhagen, Denmark; 4 Leibniz Institute of Plant Genetics and Crop Plant Research, Gatersleben, Germany; University of North Carolina at Chapel Hill, UNITED STATES

## Abstract

MicroProteins are short, single domain proteins that act by sequestering larger, multi-domain proteins into non-functional complexes. MicroProteins have been identified in plants and animals, where they are mostly involved in the regulation of developmental processes. Here we show that two *Arabidopsis thaliana* microProteins, miP1a and miP1b, physically interact with CONSTANS (CO) a potent regulator of flowering time. The miP1a/b-type microProteins evolved in dicotyledonous plants and have an additional carboxy-terminal PF(V/L)FL motif. This motif enables miP1a/b microProteins to interact with TOPLESS/TOPLESS-RELATED (TPL/TPR) proteins. Interaction of CO with miP1a/b/TPL causes late flowering due to a failure in the induction of FLOWERING LOCUS T (FT) expression under inductive long day conditions. Both *miP1a* and *miP1b* are expressed in vascular tissue, where CO and FT are active. Genetically, miP1a/b act upstream of CO thus our findings unravel a novel layer of flowering time regulation via microProtein-inhibition.

## Author Summary

MicroProteins are short single-domain proteins that possess the ability to interfere with larger multi-domain proteins. These protein species can be identified in plants and animals where they evolved from large proteins by successive domain-loss. MicroProteins often act as decoys thus interaction with their targets results in reduced activity. Here we employed a bioinformatics approach and identified a number of microProteins encoded in the Arabidopsis genome. The detailed study of two uncharacterized microProteins, miP1a and miP1b, revealed that these proteins interact with their target protein CONSTANS and additionally engage in a larger protein complex involving the co-repressor protein TOPLESS. These findings imply that miP1a/b establish a flowering activity switch similar to AUX/IAA proteins in the auxin-signaling pathway.

## Introduction

The formation of higher order protein complexes greatly expands the matrix of physiological responses and is crucial for the adjustment of developmental processes in regard to environmental changes. MicroProteins are important modulators, because they are able to prevent larger, multi-domain proteins from forming functional multimers [1–4]. It is conceivable that microProteins and their substrates exist in balanced equilibriums. Thus, microProteins can rapidly exert their full potential upon alteration of the physiological state of the cell, independently of transcription and translation.

MicroProteins are small, single domain proteins that harbor a protein-protein-interaction domain; they have the ability to engage larger, multi-domain proteins into dimers that prevent the default function of the larger protein. Several such microProteins have been identified in the past years in plants and a hallmark of all of these small proteins is their dominant negative potential [1–4]. In addition to microProteins, other protein species that are related to larger multi-domain proteins but lack distinct domains exist. We have previously coined these as “interfering proteins” [3]. According to our definition, interfering proteins are larger, often multi-domain proteins that can form complexes with other proteins but lack a certain functional domain. Although recent bioinformatics approaches have attempted to identify microProteins, they have resulted in proteins that are better suited as interfering proteins due to their large size and protein composition [5].

To identify novel microProteins, we systematically searched the *Arabidopsis thaliana* genome for transcripts encoding small, single domain proteins (see S1 Text, S1 and S2 Figs). According to our definition of microProteins, we searched for proteins fulfilling the following criteria: 1) a small size protein (here below 140 amino acids, since all of the identified true microProteins to date fall into this range); 2) contains a single protein (Pfam) domain enabling the protein to physically interact with other proteins; 3) related to larger proteins and 4) exhibits a dominant-negative mode of action. Using the first three criteria, we analyzed the Arabidopsis genome (see S1 table, S1 and S2 Figs) and *inter alia* identified two small B-Box microProteins having the potential to interact with CONSTANS (CO), a major regulator of photoperiodic flowering.

*Arabidopsis thaliana* is a facultative long day plant that flowers early when grown in long day conditions. Several genetic pathways have been characterized that act on a set of floral integrator genes, translating inputs of these different pathways into a flowering response [6]. CONSTANS (CO), the eponymous member of the family of CONSTANS-like (COL) transcriptional regulators, mediates flowering in response to photoperiod [7]. Mutations in the *CO* gene result in a late-flowering phenotype under inductive long-day conditions [8,9]. Furthermore, *CO* mRNA shows a diurnal expression pattern [10] and the stability of the CO protein is reduced in darkness [11]. *FLOWERING LOCUS T* (*FT*) is the major target of CO [12]. Both *CO* and *FT* are expressed in the vascular tissue of leaves [13]. Upon induction, the FT protein acts as a systemic signal, traveling via the phloem from the leaves to the shoot apex [14,15]. After reaching the shoot apex, FT interacts with the bZIP transcription factor FD and induces the production of the floral meristem [16,17].

Besides CO, very little is known about the function of most CO-like (COL) proteins. All COL proteins consist of one or two amino terminal B-Box domains and an additional 43 amino acid CCT-domain (CO, CO-like, TOC1-domain) at the carboxy-terminus [7,18,19]. The CCT-domain shares homology to a DNA-binding domain and might be involved in mediating protein-DNA interactions [20]. A recent study on TOC1, supports the idea that the CCT-domain binds DNA [21] and it was recently reported that CO can physically interact with the promoter of *FT* [22,23] via the CCT domain.

The B-Box zinc finger domains of CONSTANS are required for CO to be functional and several loss-of-function mutant plants have been isolated that carry mutations affecting the B-Box domains [18]. Zinc finger B-Boxes serve as protein-interaction platforms and mediate protein-protein-interactions. The type of protein-dimer CO is involved in influences CO activity and it is thought that as a homodimer, CO controls flowering by inducing expression of *FT*. Recently, it was shown that BBX19, a B-Box transcription factor of the group IV subfamily of B-Box proteins [24], when over-expressed, is able to sequester CO into a non-productive protein complex [25]. This finding illustrates that CO activity can be controlled by the type of protein complex CO is involved in.

Here we have analyzed the translated Arabidopsis ORFeome for the existence of small, single-domain proteins that based on their respective domain organization might function as microProteins that target transcriptional regulators. Our *ab initio* analysis identified a total of 44 small proteins belonging to 12 different protein families (S1, S2 Figs and S1 Table). As a proof of principle, we experimentally tested whether two small B-Box-type microProteins, we named microProtein 1a (miP1a) and miP1b, act as predicted and heterodimerize with the flowering regulator CO. Both miP1a and miP1b interact with CO in yeast, *in vitro* and *in planta*. Ectopic overexpression of either *miP1a* or *miP1b*, cause a severe delay in flowering, which is due to a strong reduction of *FT* expression. Furthermore, we show that *miP1a/b* are co-expressed with *CO* and *FT* in the vascular system and have a circadian expression profile. Both microProteins have an additional PFVFL motif that enables them to interact with TPL/TPR co-repressor proteins. We show that miP1a can mediate between CO and TPL and that the interaction with TPL is required for the late flowering phenotype of ectopic miP1a/b expression. Taken together, these findings show that attenuation of protein function by ectopic microProtein expression is a powerful tool to interfere with developmental processes and can *inter alia* be used to control the transition to flowering.

## Results

### MiP1a and miP1b are microProteins that contain a B-Box motif and interact with the flowering time regulator CONSTANS

The Arabidopsis genome contains 32 genes encoding proteins containing B-Box motifs (also called BBX proteins) [24]. Phylogenetic analysis of all Arabidopsis B-Box proteins reveals that miP1a and miP1b, are closely related to each other but cluster with both CONSTANS / CONSTANS-like proteins (S3A Fig). Interestingly, both microProtein genes are physically located in the direct vicinity of *COL* genes. These findings suggest that *miP1a/b* genes evolved during one of the genome-amplification events (whole genome duplication or tandem duplication), which enlarged the *COL* gene family (S3B Fig). Alignment of all COL B-Box domains with the B-Box domains of miP1a/b reveals that miP1a/b have one full B-Box domain and remnants of a second B-Box-domain (S3C and S3E Fig). Using structural modeling, we modeled the three-dimensional protein conformation of CO, COL6 and miP1a. These structures suggest that COL6 is more distant to CO and miP1a, which when superimposed show high degrees of structural similarities in the first B-Box domain (S3D Fig). An alignment of all COL proteins and miP1a/b further corroborates this finding and shows that critical cysteine and histidine residues of the second B-Box are also conserved in miP1a/b. These findings point towards a role of miP1a/b as potential interaction partners of COL proteins.

**Fig 1:**
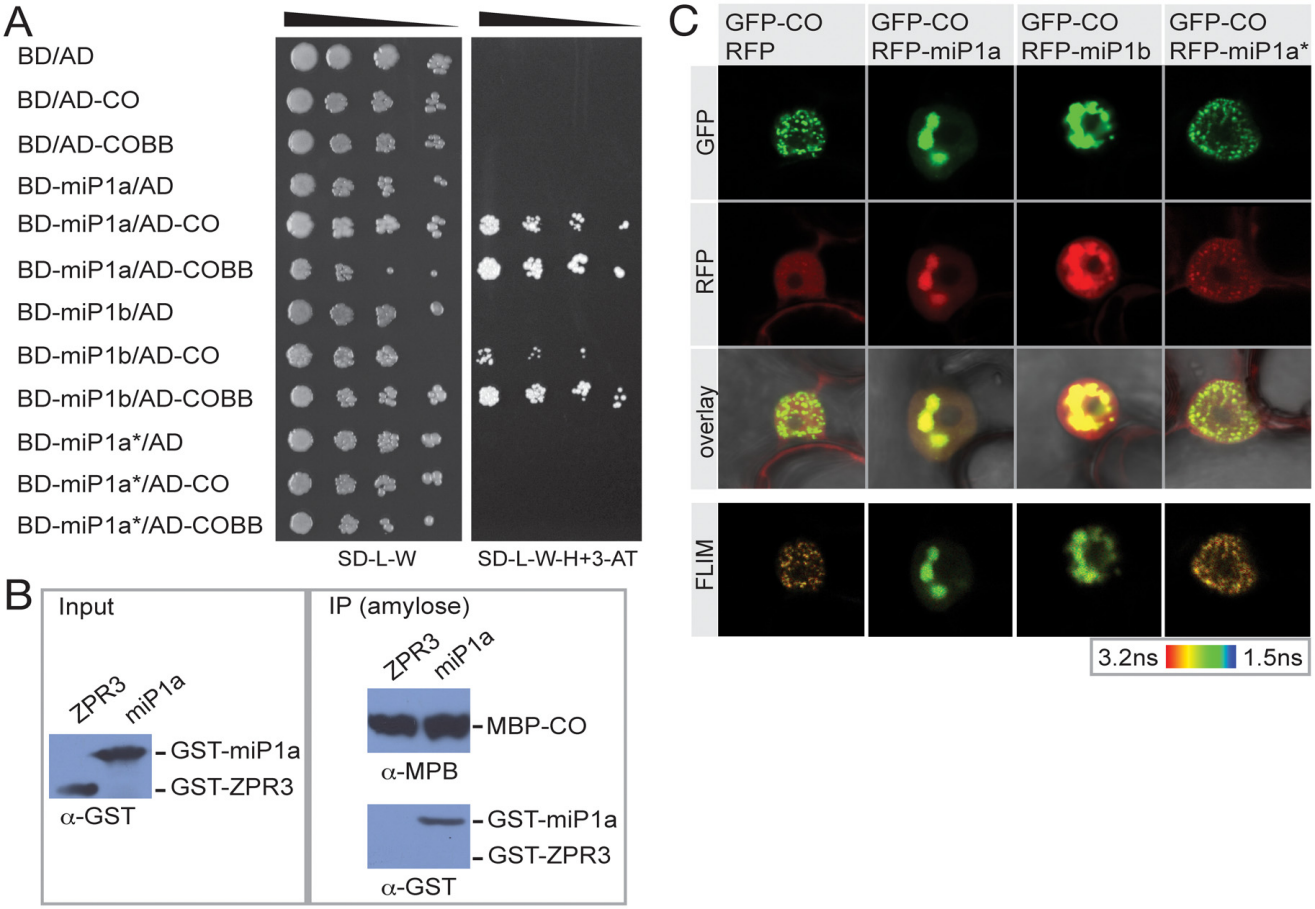
The miP1a/b microProteins physically interact with CONSTANS via the B-Box domain. (***A***) Yeast-two-hybrid interactions were tested by transforming fusions of either CO or the CO B-Box domain (CO_BB_) to the Gal4 activation domain (AD) and fusions of miP1a/b/a* to the Gal4 binding domain (BD). Growth of serial dilutions on non-selective SD-medium lacking leucine and tryptophan (-L/-W) show normal yeast growth. Only positive interactors are able to grow on restrictive growth medium supplemented with 10mM 3-Aminotriazole (3-AT) and lacking histidine. (***B***) *In vitro* pull-down experiments. Recombinant MBP-CO, GST-miP1a and GST-ZPR3 proteins were produced in *E*. *coli*. After lysis, cell extracts of either MBP-CO and GST-ZPR3 or MBP-CO and GST-miP1b were mixed and incubated with magnetic amylose beads (NEB). MBP-CO complexes were precipitated and washed using a magnetic stand, eluted by boiling in SDS-loading buffer and separated by SDS-PAGE. Proteins were detected by immunoblotting. (***C***) Transient co-expression of fluorescently labeled proteins in *Nicotiana benthamiana* leaves. GFP:CO localizes to sub-nuclear speckles and in the case of co-transformation with RFP:miP1a and RFP:miP1b, co-localization in speckles is observed. FLIM images show significant changes in the life-time of GFP when functional microProteins are co-transformed. GFP life-times of co-transformations of RFP:miP1a* resemble the RFP controls.

Based on the structure of the B-Box domains of both miP1a/b and CO, we postulated that miP1a/b function by forming heterodimeric complexes, that sequester CO/CO-like (COL) proteins into non-functional complexes. To test whether CO physically interacts with miP1a/b, we performed directed yeast-two-hybrid studies. The coding sequences of CO and the B-Boxes of CO were fused in frame to the Gal4-activation domain (AD; pGADT7) and used as prey. The prey proteins were tested in yeast against the empty pGBKT7 vector expressing the Gal4-DNA binding domain (BD) and in frame fusions of miP1a, miP1b and miP1a*, miP1a* being a protein in which all cysteine and histidine residues of the B-Box were mutated to alanine to prevent dimerization. We observed that CO and the CO B-Box-domain are able to interact with both miP1a and miP1b in yeast (Fig 1A). As predicted, no interaction was observed with the miP1a* protein, confirming that an intact zinc finger B-Box is essential for this interaction.

To verify that the interactions of miP1a/b with CO, which were initially observed in yeast, can also occur in a different system, we tested if miP1a and CO expressed and purified from *E*. *coli* cells, can be co-immunoprecipitated. We expressed fusions of CO to the maltose binding protein (MBP) and fusions of miP1a to the glutathion-S-transferase tag (GST). As a negative control we fused the LITTLE ZIPPER3 (ZPR3) protein, a small leucine-zipper microProtein to GST-tag. All fusion proteins were expressed under the inducible T7 promoter in *E*. *coli* BL21 cells. After cell lysis, soluble protein fractions of either GST-miP1a and MBP-CO or GST-ZPR3 and MBP-CO were mixed and incubated with amylose-coated magnetic beads. After precipitation and washing, immune complexes were released by boiling in SDS-loading buffer and separated by SDS-PAGE. CO was able to physically interact with the miP1a microProtein (Fig 1B) whereas no binding of GST-ZPR3 to MBP-CO was observed (Fig 1B). This further supports the idea that miP1-type microProteins act by binding to the CO protein and that this binding does not require other accessory proteins.

Potential inhibition of CO by miP1a/b could either be as a result of preventing the CO protein from entering the nucleus, or by attenuating DNA-binding of CO. To determine if miP1a/b can retain CO in the cytoplasm, we transiently co-transformed tobacco leaves with fusions of CO to the green fluorescent protein (GFP) and fusions of miP1a, miP1b and miP1a* to the red fluorescent protein (RFP). We observe that both miP1a and CO and miP1b and CO co-localize in the same sub-nuclear structures (Fig 1C). Little fluorescence is observed in the cytoplasm, excluding the possibility that miP1a/b act by preventing nuclear import of CO. To test whether CO and miP1a/b also physically interact *in planta*, we performed FRET/FLIM experiments and detected significant lifetime changes of the GFP fluorophore in the speckles in which CO and miP1a/b co-localize (Fig 1C and S4 Fig). No significant lifetime changes were observed in nuclei co-expressing free RFP or RFP-miP1a*. Taken together, these results demonstrate that miP1a/b and CO are able to physically interact *in planta* through their B-Box domains and that these interactions do not inhibit nuclear localization of CO.

### Overexpression of either miP1a or miP1b delays flowering under inductive long day conditions

**Fig 2:**
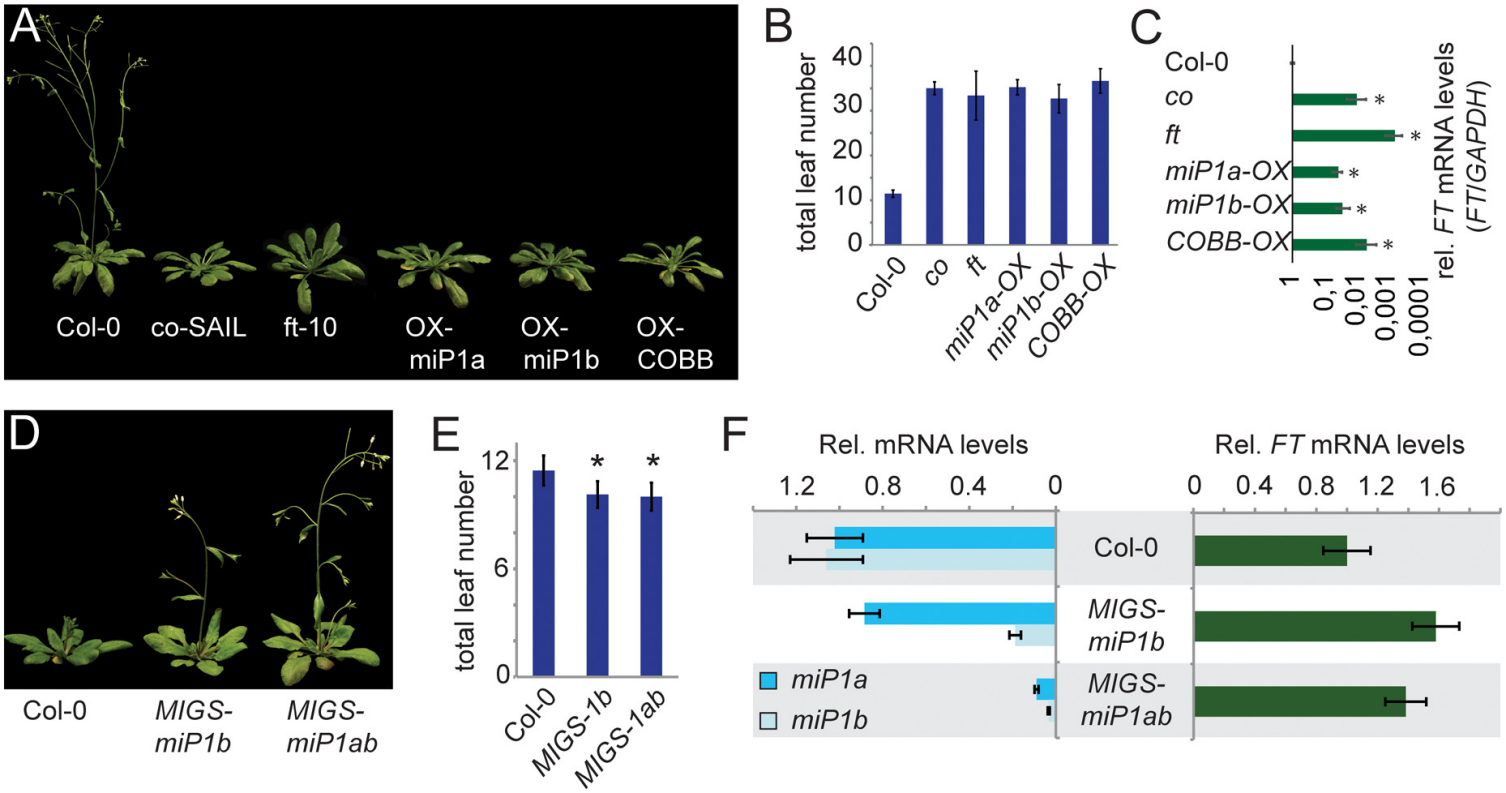
Transgenic plants with elevated microProtein levels are late flowering under inductive long day conditions owing to decreased levels of *FT* expression. (***A***) Image of representative late flowering *co*, *ft*, *35S*::*miP1a*, *35S*::*miP1b* and *35S*::*CO*_*BB*_ plants compared to a Col-0 wild type plant of the same age. (***B***) Quantification of flowering in long day conditions by counting the number of leaves produced at bolting. Error bars represent the standard deviation. (***C***) Quantification of transcript levels by qRT-PCR shows that *FT* expression levels in all late flowering plants are severely reduced compared to wild type Col-0 plants. Asterisk p<0.001. (***D***) Image of representative early flowering *35S*::*MIGSmiP1b* and *35S*::*MIGSmiP1ab* transgenic plants compared to a Col-0 wild type plant of the same age. (***E***) Quantification of flowering in long day conditions by counting the number of leaves produced at bolting. Error bars represent the standard error. Asterisk p<0.01. (***F***) Quantification of transcript levels by qRT-PCR shows that *miP1b* mRNA expression levels are significantly reduced in *35S*::*MIGS-miP1b* transgenic plants while *FT* mRNA levels are slightly increased. In *35S*::*MIGS-miP1ab* transgenic plants expression levels of both miP1 and miP1b mRNA are strongly reduced compared to Col-0 wild type plants while *FT* mRNA levels are slightly increased.

To experimentally test the hypothesis that overexpression of miP1-type microProteins would have a dominant-negative effect on its predicted target CO we overexpressed the *miP1a* and *miP1b* genes. The coding sequences of miP1a/b were isolated by PCR and recombined in the pJAN33 vector [26] harboring a tandem-*CaMV35S* promoter for high-level ectopic overexpression. For each construct (*pJAN33-miP1a* and *pJAN33-miP1b*), we isolated (15 and 25 respectively) individual T1 transgenic lines that showed resistance to the herbicide BASTA respectively. The majority (about 80%) of the recovered transgenic plants showed severely delayed flowering when grown in long day conditions. To exclude an effect of the herbicide BASTA, we selected three independent homozygote transgenic lines and tested the flowering behavior under controlled inductive long day conditions. This analysis revealed that the transition to flowering of transgenic microProtein-overexpression plants is extremely compromised under inductive long-day condition when compared to wild type Col-0 plants (Fig 2A and 2B). Interestingly, the *CO* locus produces an alternatively spliced transcript, which could potentially produce a protein with only the B-Box domains. Overexpression of this CO splice variant (CO_BB_) resulted in a similar late flowering phenotype. Furthermore, overexpression of *miP1a/b* and *CO*_*BB*_ caused a severe decrease in the levels of *FT* mRNA in leaves of long day grown plants (Fig 2C), explaining the molecular nature of the observed late flowering phenotypes. Phenotypically and molecularly, *miP1a/b* overexpression plants strongly resemble plants carrying loss-of-function mutations in either the *CO* or *FT* gene. These findings support our predictions and indicate that ectopic expression of miP1-type microProteins renders CO non-functional, resulting in attenuation of *FT* expression, which seems causal for the observed late flowering phenotypes. Overexpression of the mutant miP1a* protein does not cause an alteration in the flowering behavior of transgenic plants (S5 Fig), indicating that a functional zinc-finger B-Box domain is required for the observed late flowering phenotype of miP1a. Overexpression of CO_BB_, miP1a or miP1b did also not cause flowering time changes when transgenic plants were grown under short day conditions (S6 Fig). Since CO is inactive in short days, our findings suggest that the most likely mode of miP1a/b action is rendering CO inactive in long day conditions and further suggest that miP1a/b affect CO and not other flowering-promoting factors. Further support that miP1a/b act through CO is provided by the finding that ectopic application of the flowering promoting hormone gibberellic acid (GA) induces flowering in *mip1a/b-OX* transgenic plants to the same extend as in *co* mutant plants (S7 Fig). These findings indicate that the two microProteins do not affect the autonomous regulatory pathway and other meristem factors that can be induced by GA [27].

Finally, *co* loss-of function mutants with ectopic *miP1a* expression (*co miP1a-OX*) are indistinguishable from either *miP1a-OX* or *co* mutant plants in their flowering behavior (S8 Fig), implying that CO is required for the flower attenuating effect of miP1a and that miP1a/b do not function by controlling other pathways.

### Specificity of the interaction of miP1a/b with CONSTANS

Both miP1a and miP1b proteins have a B-Box zinc finger domain allowing them to interact with CONSTANS and potentially with the many other proteins containing a similar B-Box domain (B-Box proteins, BBX proteins). To further investigate the possible use of B-Box proteins as modulators of flowering time, we overexpressed B-Box proteins of group II (COL9), group III (COL16) and group IV (STO); we also included artificial microProtein versions (COL9miP, COL16miP, STOmiP) encoding only the respective B-Box domains. The initial analysis of T1 transgenic plants revealed that none of these transgenic lines was able to significantly promote or delay the floral transition (S9 Fig).

We have also attempted to study the effect of lost *miP1a/b* activity using available T-DNA insertion lines and transgenic plants overexpressing artificial microRNAs. Owing to the small size of genes encoding microProteins, T-DNA insertions in microProtein genes are more infrequent compared to larger genes. We have characterized the only available T-DNA insertion line in the *miP1a* gene (GABI-KAT line *288G08*). This line however did not show a reduction or loss of *miP1a* mRNA levels but had slightly increased levels of miP1a expression; flowering time was comparable to wild type plants (S10A and S10B Fig). Transgenic plants overexpressing artificial microRNAs targeting both *miP1a* and *miP1b* also neither showed a mutant phenotype nor were *miP1a/b* mRNA levels substantially decreased (S10C Fig).

To study the flowering behavior of plants with reduced *miP1a/b* mRNA levels we used the microRNA-induced gene silencing (MIGS) technology [28] and overexpressed the sequences encoding the miP1a/b-specific carboxy terminal regions (for *MIGS-miP1a* and *MIGS-miP1b*) or the full-length coding sequences of both *miP1a* and *miP1b* fused to a *miR173*-binding site (for *MIGS-miP1ab*). These fusion constructs are recognized by *miR173*, which elicits the production of trans-acting siRNAs (*tasi-RNAs*) that target then either *miP1a* or *miP1b* alone or *miP1a* and *miP1b* mRNA simultaneously. In total we were able to recover two transgenic plants overexpressing a *miP1a-MIGS* construct and ten transgenic plants overexpressing a *miP1b-MIGS* construct. Both *miP1a-MIGS* transgenic plants exhibited wild type flowering behavior whereas six out of ten *miP1b-MIGS* were slightly early flowering. Because this flowering time phenotype was weak, we performed a double-blind flowering time study of progeny plants of one representative line in long day conditions. In this experiment *miP1b-MIGS* transgenic plants flowered slightly but significantly earlier compared to Col-0 wild type plants (Fig 2D and 2E). In addition, we also found *miP1b* mRNA levels to be significantly reduced and *FT* mRNA to be slightly increased in expression (Fig 2F). It is interesting to note that *miP1a* expression is not significantly affected by overexpression of the *MIGS-miP1b* construct, suggesting that there is little cross-reactivity of the *tasiRNAs*. In addition to *MIGS-miP1b* transgenic plants, we also generated transgenic *MIGS-miP1a/b* plants by overexpressing their respective coding sequences fused to the *miR173* target sequence. *MIGS-miP1a/b* transgenic plants also exhibited an early flowering phenotype under long day conditions; had strongly reduced levels of both *miP1a* and *miP1b* and slightly increased levels of *FT* mRNA. These findings suggest that miP1b, and maybe to a lesser extend miP1a, play a role in the CO-mediated long-day flowering-promotion pathway.

Besides flowering time control B-Box proteins have been described to control other developmental and adaptive growth processes. For example when overexpressed, STO can e.g. promote root growth in high salt conditions [29]. Using the same growth conditions, we tested whether besides flowering time control miP1a/b might have additional roles when ectopically expressed. In response to high salt concentrations neither *miP1a* nor *miP1b* had a significant effect on root elongation growth when overexpressed (S11 Fig) supporting the idea that the major role of miP1a/b seems to be flowering time control.

### The diurnal pattern of *miP1a/b* mRNA expression partially coincides with *CO* mRNA expression peaks

**Fig 3:**
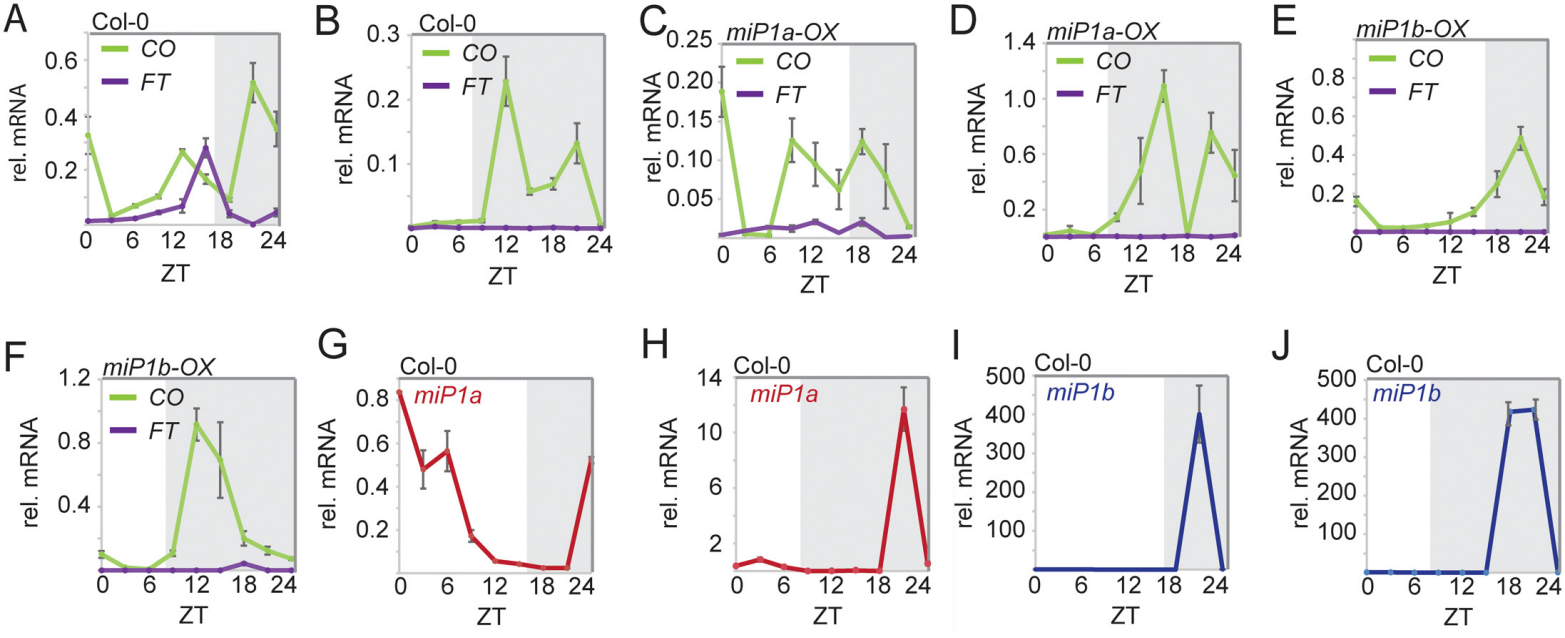
Diurnal expression profiles of *CO*, *FT*, *miP1a* and *miP1b*. Quantitative RT-PCR analysis of *CO* and *FT* (***A-F***), *miP1a* (***G*,*H***) and *miP1b* (**I,J**). Plants were grown in 16-hour long days (***A*, *C*, *E*, *G*, *H***) or 8-hour short day conditions (***B*, *D*, *F*, *H*, *J***). Samples were harvested every 3 h over a time period of 24 h. Expression levels are relative to *GAPDH* and the error bars represent the standard deviation of four technical replicates. (***A*,*B***) *CO* and *FT* expression in Col-0 wild type plants. (***C*,*D***) *CO* and *FT* expression in transgenic *35S*::*FLAG-miP1a* plants. (***E*,*F***) *CO* and *FT* expression in transgenic *35S*::*FLAG-miP1b* plants. (***G*,*H***) Expression profile of *miP1a* in LD and SD. (***I*,*J***) Expression profile of *miP1b* in LD and SD.

The abundance of *CO* mRNA is regulated by GIGANTEA and exhibits a diurnal expression pattern with a peak of expression occurring 16 hours after dawn both in long and short day conditions [10]. Following the peak of *CO* mRNA, *FT* mRNA expression also increases, which is causal for the early flowering phenotypes of Col-0 wild type plants in long day conditions (Fig 3A). Under short day conditions only *CO* mRNA shows a cyclic expression pattern, while *FT* mRNA is not induced (Fig 3B). When *miP1a* is ectopically expressed at high levels, *CO* mRNA abundance remains largely unchanged while the expression levels of *FT* mRNA lack the typical peak in expression at the end of the long day (Fig 3C and 3D). High ectopic expression of *miP1b* also caused changes to the circadian expression profile of *CO* mRNA and the peak towards the end of the light period was absent (Fig 3E and 3F). However, ectopic expression of either *miP1a* or *miP1b* resulted in non-cyclic *FT* expression in response to long day conditions, which supports the late flowering phenotype of the respective transgenic plants.

To test whether *miP1a/b* exhibit diurnal mRNA expression profiles similar to that of *CO*, we tested their expression by qRT-PCR. We detected diurnal patterns of expression for the mRNAs of both microProtein genes with a maximum expression towards the end of the 24-hour period (Fig 3G–3K). The *miP1a* mRNA also showed a second peak of expression in the early time points of long days (Fig 3G). It is also interesting to note that *miP1a* mRNA levels are higher in short days compared to long days. The expression pattern of *miP1b* is highly reminiscent of the *CO* diurnal mRNA profile under both short and long days (Fig 3I and 3K).

Taken together, we demonstrated that both microProtein genes show diurnal mRNA expression profiles with expression maxima coinciding with elevated levels of *CO* mRNA. When *miP1a/b* are overexpressed *CO* mRNA remains largely unchanged while *FT* mRNA is no longer up-regulated towards the end of the long day photoperiod. This finding supports the idea that CO protein activity is affected when miP1a/b are ectopically high.

### Overexpression of *miP1a* in transgenic plants ectopically expressing *CO* alters flowering time

**Fig 4:**
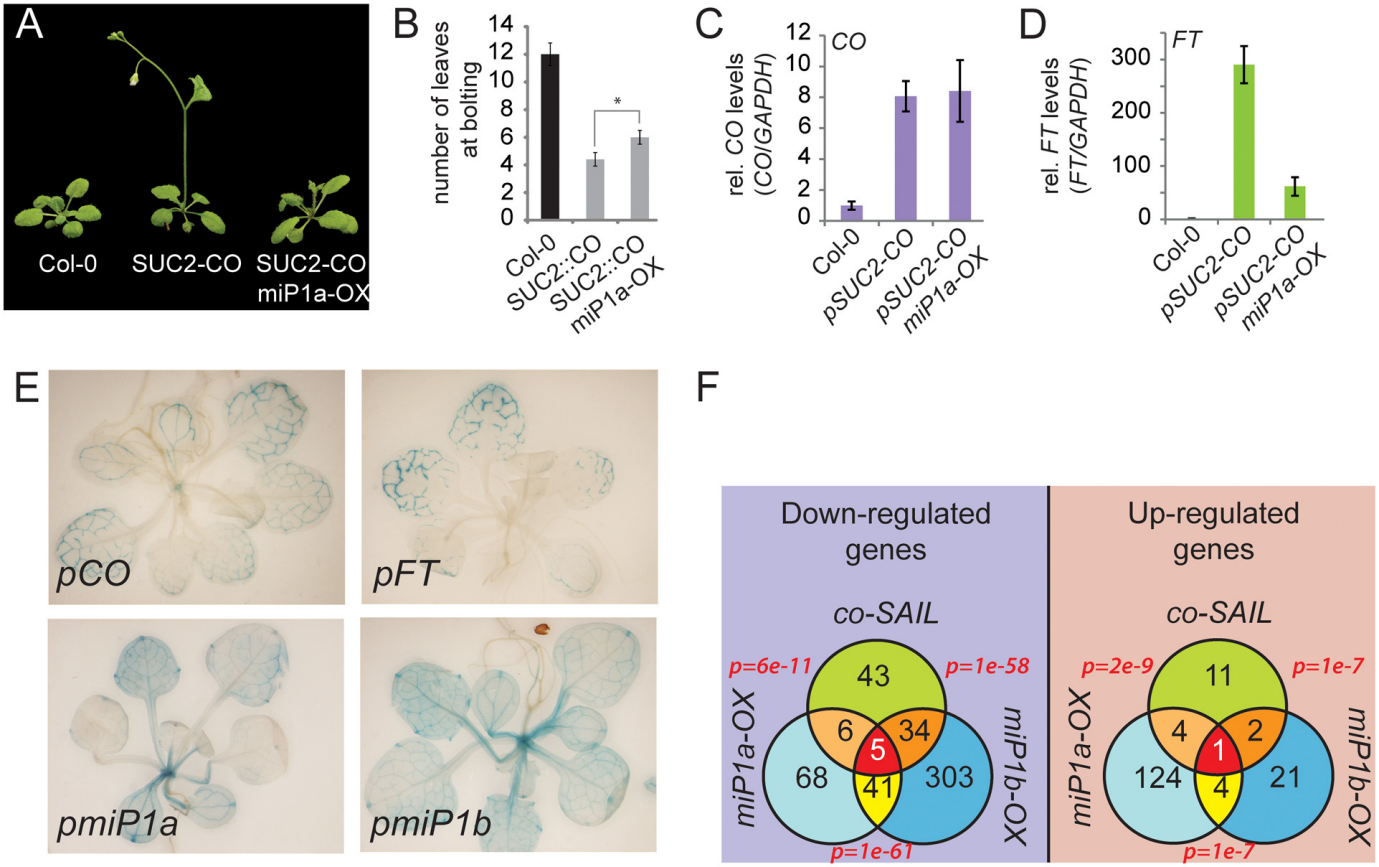
Genetic interaction studies reveal that miP1a/b control CO protein activity, have the same spatial expression pattern and are feedback-regulated via the photoperiodic flowering time pathway. (***A***) Image of representative Col-0 wild type, *SUC2*::*CO* and *SUC2*::*CO 35S*::*miP1a* plants of the same age. (***B***) Quantification of flowering in long day conditions by counting the number of leaves produced at bolting. Asterisk p<0.001. (*c*, *d*) Quantification of transcript levels of (***C***) *CO* and (***D***) *FT* by qRT-PCR shows that *CO* expression levels are up-regulated in both *SUC2*::*CO* and *SUC2*::*CO 35S-miP1a* plants but *FT* levels are higher in *SUC2*::*CO* compared to *SUC2*::*CO 35S-miP1a*. (***E***) Spatial expression pattern of *CO*, *FT* and *miP1a* and *miP1b* reveals that all genes are expressed in vascular tissue of vegetative rosette leaves. (***F***) Comparison of the transcriptomes of *co-SAIL*, *35S*::*FLAG-miP1a* and *35S*::*FLAG-miP1b* relative to Col-0 wild type plants. Venn diagrams showing overlap between up- and down-regulated genes; p-values are based on the hypergeometric distribution function (phyper using R 3.2.2).

To assess whether miP1a/b have the potential to inhibit CO activity in transgenic plants overexpressing CO in the vasculature, we crossed very early flowering *SUC2*::*CO* plants with late flowering *35S*::*miP1a* plants. Progeny plants carrying both transgenes show an intermediate flowering behavior when compared to wild type and *SUC2*::*CO* plants (Fig 4A and 4B). This delay in flowering is not due to an effect on the levels of *CO* expression (Fig 4C). However, *FT* levels are significantly lower in *SUC2*::*CO 35S*::*miP1a* plants compared to *SUC2*::*CO* plants explaining the intermediate flowering phenotype (Fig 4D). When compared to wild type plants, the levels of *FT* expression in *SUC2*::*CO 35S*::*miP1a* plants are still strongly induced (around 50-fold). This can be explained by the fact that the *SUC2* promoter is much stronger in the phloem companion cells than the *35S* promoter and thus CO is more abundant causing *SUC2*::*CO 35S*::*miP1a* plants to flower earlier than the wild type.

### The microProteins *miP1a/b* are expressed in the vasculature of leaves

Analysis of the spatial expression patterns of both *CO* and *FT* revealed that they are expressed in the vasculature of leaves [30]. Expression of both genes in vascular cells is also sufficient to trigger the transition to flowering [13]. We attempted to investigate the spatial expression patterns of miP1-type microProteins. Expression analysis of *miP1a* and *miP1b* in transgenic plants expressing a genomic fragment of either *miP1a* or *miP1b* fused to the beta-glucuronidase gene (GUS), revealed that both microProteins have a broader and more patchy pattern of expression compared to *CO* but are also predominantly expressed in vascular tissue (Fig 4E) of leaves, the tissue where CO is acting to regulate photoperiod-dependent flowering.

In addition to the expression in leaves we also detected GUS expression for both *miP1a/b* in petioles of leaves where *CO* does not seem to be expressed. It is interesting to note that both microProtein genes are highly abundant in the shoot apical meristem, where *CO* also seems to be expressed.

The finding that *miP1a/b* are co-expressed in vascular tissue and have the ability to interact with CO, supports a regulatory role. Furthermore, when ectopically expressed in the phloem companion cells, *SUC2*::*miP1a* can also strongly delay the floral transition indicating that miP1a is functional in the phloem and that CO is likely the major target of miP1a (S12 Fig).

### Identification of transcripts affected by CO inactivation

To further corroborate the idea that the predominant function of miP1a/b is to regulate CO protein activity, we characterized transcriptomes of Col-0 wild type, *co* mutants (*co-SAIL*) and the transgenic plants overexpressing *miP1a* and *miP1b* using RNA-Seq (see S1 Data). The downregulated-transcriptomes of *35S*::*FLAG-miP1a* and *35S*::*FLAG-miP1b* have a 60% overlap which is quite substantial but not surprising. Interestingly, around 80% of the genes down-regulated in the *co* mutant background (relative to Col-0) are also down-regulated in the transgenic *35S*::*FLAG-miP1b* plants (Fig 4F) supporting the idea that CO protein activity is strongly compromised by miP1b-overexpression. To validate the observation that differentially expressed genes identified by mRNA-Seq are truly altered in expression, we performed individual qRT-PCRs to test expression of five candidate genes (S13 Fig). These RT-PCRs largely confirm the RNA-Seq results. We find genes down-regulated in all three genotypes (e.g. *FUL* and *At3g49340*) but also genes whose expression is unchanged in *35S*::*FLAG-miP1a* but down-regulated in *co* mutants and *35S*::*FLAG-miP1b* (e.g. *ZAT7*) indicating that miP1a and miP1b might also have diverging functions. The same is true for genes up-regulated in the investigated genotypes (S13 Fig). In all three genotypes (*co*, *35S*::*FLAG-miP1a*, *35S*::*FLAG-miP1b*), the expression levels of *FT* are among the top down-regulated genes confirming that the late flowering phenotype of *35S*::*FLAG-miP1a* and *35S*::*FLAG-miP1b*, like in *co* mutants, is due to the failure of inducing *FT* expression. Another flowering time gene found to be down-regulated in all three genotypes is *FRUITFUL* (*FUL*) which acts downstream of FT [31], further supporting the hypothesis that miP1a/b act by inhibiting CO activity. These findings are in agreement with unchanged *CO* mRNA levels in *miP1a/b* over-expression plants, which indicates that the inhibition of CO likely occurs at the post-translational level. We also analyzed genes up-regulated in *co*, *35S*::*FLAG-miP1a*, *35S*::*FLAG-miP1b* and found *MADS AFFECTING FLOWERING5* (*MAF5*) to be up-regulated in all three genotypes relative to Col-0 (Fig 4F). MAF5 acts as a floral repressor that is strongly controlled epigenetically [32,33], which is in line with the late flowering phenotype observed in *co* loss-of-function and *miP1a/b* gain-of-function plants. Whether and how elevated *MAF5* mRNA levels contribute to the late flowering phenotype of *co* mutant plants is currently unknown.

### Phylogenetic analysis of miP1a/b-type microProteins in different plant genomes

**Fig 5:**
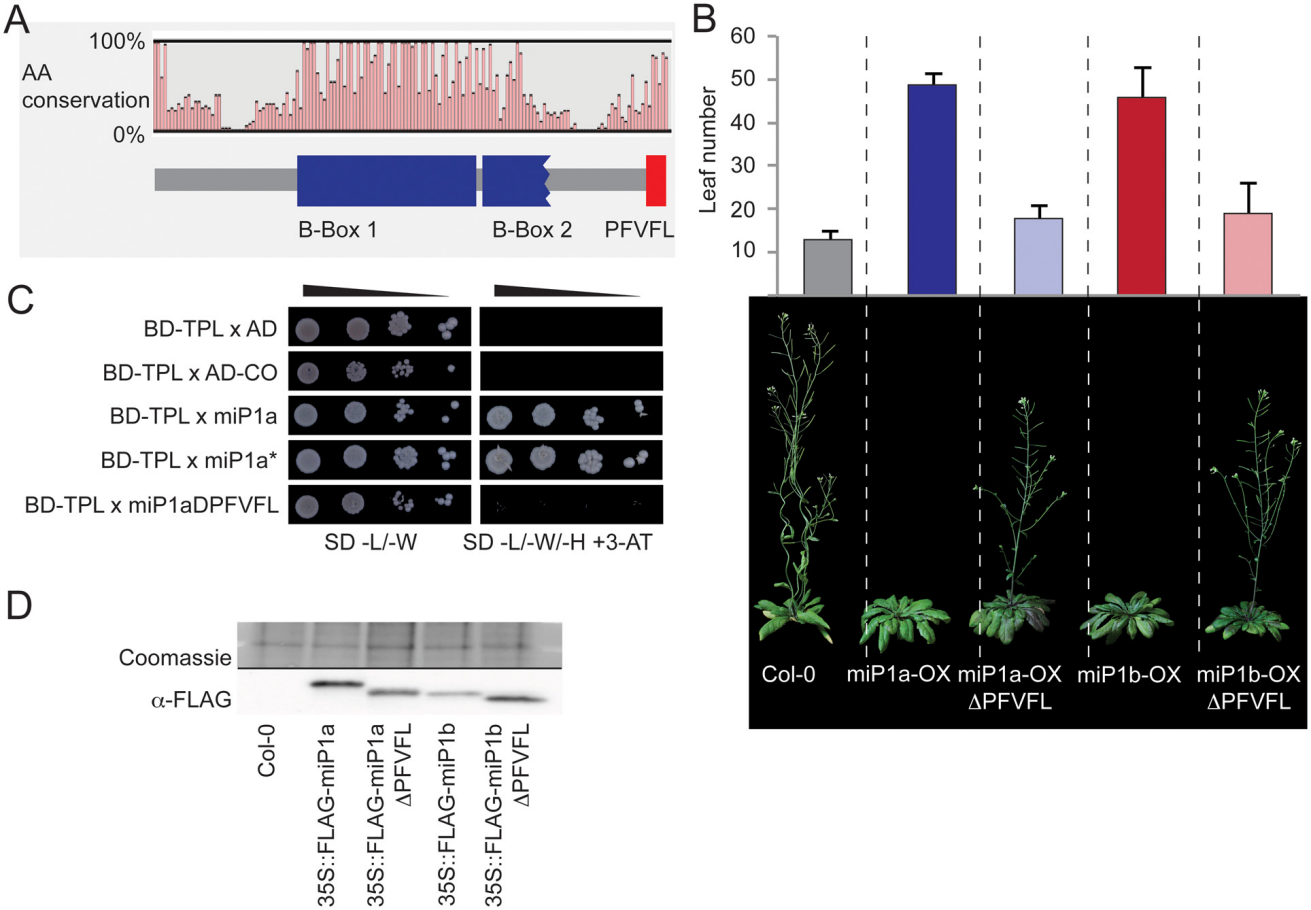
The microProteins miP1a/b interact with the TOPLESS co-repressor protein to repress flowering. (***A***) Summary of the multiple sequence alignment of miP1a/b proteins from different dicotyledonous species. Cartoon depicts the B-Box domains and the carboxyterminal PF(V/L)FL motif. (***B***) Upper panel: Quantification of flowering in long day conditions by counting the number of leaves produced at bolting. Plotted are average leaf numbers of at least 10 individual plants and error bars represent the standard deviation. Lower panel: Image of representative late flowering *35S*:*FLAG*:*miP1a* and *35S*::*FLAG*:*miP1b* transgenic plants compared to a Col-0 wild type plant of the same age and two lines of *35S*:*FLAG*:*miP1a*Δ*PFVFL* and *35S*::*FLAG*:*miP1b*Δ*PFVFL* showing an intermediate flowering behavior. (***C***) Yeast-two-hybrid interaction of miP1a with the TOPLESS co-repressor. Growth of serial dilutions on non-selective SD-medium lacking leucine and tryptophan (-L/-W) show normal yeast growth. Only positive interactors were able to grow on restrictive growth medium supplemented with 10mM 3-Aminotriazole (3-AT) and lacking histidine. (***D***) Western blot analysis of transgenic plants.

To gain more information on how miP1a/b-type proteins have evolved, we used Phytozome [34] and extracted all miP1a/b-type related proteins from different species. The multiple sequence alignment of all species revealed that the first B-Box and the remnants of the second B-Box are highly conserved. Surprisingly, there is a very high conservation for the last five amino acids, constituting the PF(V/L)FL motif (Fig 5A and S14 Fig). Phylogenetic analysis revealed that miP1a/b-type proteins evolved in the *Pentapetalae* family of dicotyledonous plants. Using the last five amino acids as anchor, we find that the carboxy terminal motif of the most ancient miP1a/b-type proteins in the *Fabidae* family varies significantly (S15 and S16 Figs). In *Glycine max* for example, we find one protein with the sequence LSLLL that strongly resembles the LxLxL motif, which has been shown to mediate interactions with TOPLESS-related co-repressor proteins. It is interesting to note that the PFVFL motif that is found exclusively in the *Brassicaceae* family evolved by acquiring a single point mutation that changed the leucine in the middle position to a valine. Because of the high degree of conservation of the PF(V/L)FL motif, we can assume that it confers a biological activity to miP1a/b-type proteins. The finding that the ancestral motif strongly resembles a TOPLESS-interaction motif suggested to us that these small proteins might function by engaging with TOPLESS/TOPLESS-related co-repressor proteins.

### MiP1a/b act by recruiting TOPLESS co-repressor proteins

The analysis of publicly available protein interactome data [35] further indicated that miP1a could potentially interact with TOPLESS/TOPLESS-RELATED co-repressor proteins. To test if miP1a/b type microProteins interact with TOPLESS (TPL), we performed direct yeast-two-hybrid interaction test. In this assay both miP1a and miP1*, the latter having mutations in the B-Box domain, interacted with the TPL protein (Fig 5C). CO protein did not interact with TPL in this assay and neither did miP1aΔPFVFL, a miP1a variant lacking the last five amino acids (Fig 5C). To further explore the possibility that the PF(V/L)FL motif has an *in vivo* function, we compared transgenic plants overexpressing either full-length miP1a/b proteins with transgenic plants overexpressing protein variants lacking the last five amino acids (*35S*::*FLAG*:*miPa/b*Δ*PFVFL*). Under inductive long day conditions, both miP1a/b overexpressors exhibit a late flowering phenotype whereas transgenic plants overexpressing either miP1aΔPFVFL or miP1bΔPFVFL exhibit an intermediate flowering behavior (Fig 5B). To exclude the possibility that these transgenic plants accumulate diverging amounts of miP1a/b proteins we determined protein expression levels by western blot analysis. We find that the levels of transgenic proteins are largely similar (Fig 5D) excluding the possibility that removal of the PF(V/L)FL motif affects transcript or protein stability.

**Fig 6:**
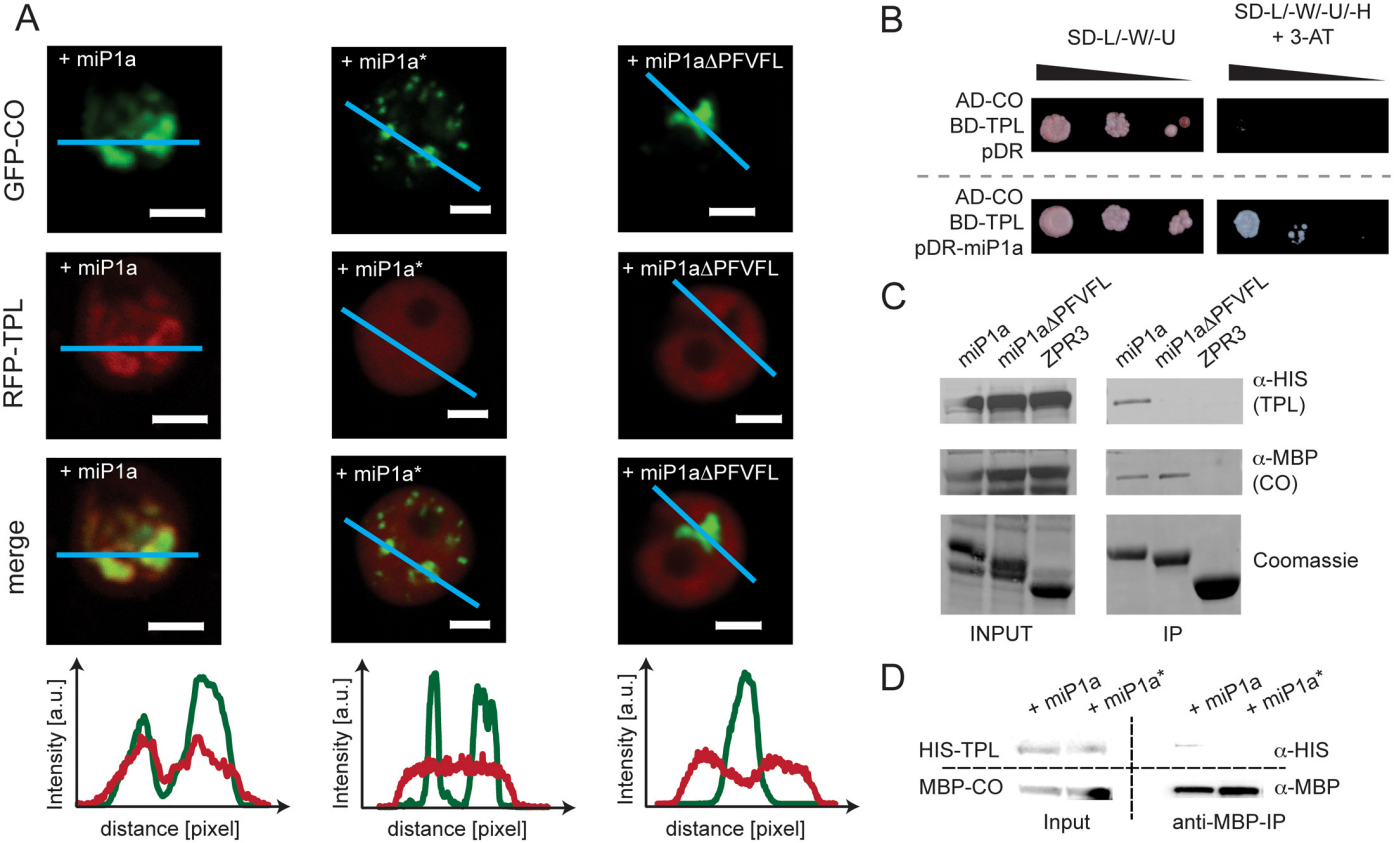
The microProteins miP1a/b act by engaging CO in a TOPLESS/TOPLESS-like co-repressor complex. (***A***) Representative image series of co-localization studies of GFP-CO and RFP-TPL co transformed with either miP1a (n = 15), the B-Box-dead version miP1a* (n = 16) or miP1aΔPFVFL (n = 9) that is lacking the TPL-interaction motif. (***B***) Yeast-three-hybrid demonstrating the formation of a CO-TPL-miP1a trimeric complex. Growth of serial dilutions on non-selective SD-medium lacking leucine, tryptophan and uracil (-L/-W/-U) show normal yeast growth. Only positive interactions were able to grow on restrictive growth medium supplemented with 10mM 3-Aminotriazole (3-AT) and lacking histidine. (***C***) *In vitro* pull-down experiments. Recombinant MBP-CO, GST-miP1a, GST-miP1aΔPFVFL, GST-ZPR3 and HIS-TPL proteins were produced in *E*. *coli*. After cell lysis, cell extracts of MBP-CO and HIS-TPL were mixed with GST-miP1a, GST-miP1aΔPFVFL or GST-ZPR3 and incubated with magnetic anti-GST coupled magnetic beads (Promega). GST-miP1a, GST-miP1aΔPFVFL and GST-ZPR3 complexes were precipitated and washed using a magnetic stand, eluted by boiling in SDS-loading buffer and separated by SDS-PAGE. HIS-TPL and MBP-CO Proteins were detected by immunoblotting. (***D***) *In vitro* pull-down experiment of the trimeric TPL-miP1a-CO complex. MBP-CO and HIS-TPL were mixed with either miP1a or miP1a* proteins. After immunoprecipitation of MBP-CO with an amylose resin proteins were detected by immunoblotting.

When GFP-CO and RFP-TPL are transiently co-expressed with either miP1a, miP1a* (B-Box dead mutant that does not interact with CO) or miP1aΔPFVFL (miP1a version lacking TPL-interaction motif) different sub-nuclear localizations pattern can be observed (Fig 6A). RFP-TPL shows a high degree of co-localization with GFP-CO, when the wild type miP1a version is co-expressed. Co-expression of the miP1a* mutant version results in an even distribution of RFP-TPL throughout the nucleus while CO-GFP localizes in small sub-nuclear speckles. We observe large sub-nuclear speckles for GFP-CO when either miP1a or the miP1aΔPFVFL variant is co-expressed. However, in the case of miP1aΔPFVFL, we see exclusion of RFP-TPL from the CO speckles implying that miP1a/b-type microProteins engage CO and TPL in a tripartite complex.

Because miP1a/b-type microProteins do not harbor a DNA-binding motif it seems likely that they act as adaptors to recruit TPL/TPR co-repressor proteins to transcription factors and bridge between the transcription factor and the co-repressor complex. To investigate this hypothesis we performed a yeast-three-hybrid study and tested whether miP1a is able to bridge between CO and TPL, which showed no interaction in the yeast-two-hybrid system (Fig 6B). When co-transformed with the empty pDR plasmid, AD-CO and BD-TPL were still unable to induce yeast growth on selective medium. However, in the presence of the miP1a protein, yeast growth was strongly induced, supporting the idea that miP1a is able to bridge between CO and TPL. These findings support the idea that miP1a/b-type proteins act as TPL/TPR-bridging factors for B-Box transcription factors and engage these transcription factors in transcriptional repressor complexes. The existence of a CO-miP1a-TPL trimeric complex is further supported by *in vitro* pull down assays, where MBP-CO and 6xHis-TPL can be co-precipitated with GST-miP1a (Fig 6C). GST-miP1aΔPFVFL is able to bind MBP-CO but fails to bind the 6xHis-TPL protein. GST-ZPR3 was used as negative control and can neither bind MBP-CO nor 6xHis-TPL. Experiments with MBP-CO as bait for HIS-TPL confirm this observation, as TPL only co-precipitates when wild type miP1a protein is present. The presence of miP1a* which cannot interact with CO is unable to facilitate an interaction between CO and TPL. Taken together, these experiments demonstrate that miP1a/b-type microProteins can mediate between CO and the transcriptional repressor TPL.

## Discussion

Several microProteins have been identified in plants in the past years. A commonality among all of these proteins is the ability to trap larger, multi-domain proteins into non-productive heterodimeric complexes. Our study reveals a number of naturally occurring small proteins that have a protein-protein-interaction domain and might interfere with larger multi-domain proteins by controlling protein activity (S1A, S1B Fig and S1 Table).

### CO activity can be regulated by the formation of different types of protein complexes

Transcription factors are often organized in gene families and the type of complexes they engage in can strongly modulate their activities. For CO it was recently shown that interaction with the BBX19 transcription factor renders CO non-functional [25]. Here we show that the transition to flowering, a trait controlled by the CO protein, can also be attenuated by overexpressing naturally occurring miP1a/b-type microProteins. MiP1a/b-type microProteins not only sequester CO but also engage it into a TPL/TPR repressor complex (Fig 5). Depletion of TPL/TPR from the CO/miP heterodimeric complex by removing the PFVFL-motif alleviates flowering time further suggesting that miP1a/b interact with CO only weakly and the interaction is stabilized by TPL/TPR proteins.

TPL/TPR proteins have a known role in flowering time control through the interaction with AP2-like transcription factors that harbor classical TPL-interaction motifs (EAR-domain). TOE1, one of these AP2-likes, acts as floral repressor [36,37]. TOE1 overexpression causes late flowering by reducing the levels of *FT*, whereas a mutation in the *TPL* gene cause slightly early flowering plants [38]. However, due to the pleiotropic nature of the *tpl* loss-of-function mutant and higher order *tpl tpr* mutants, it seems that several repressor proteins are affected in their function. For TPL-adapter proteins such as miP1a/b, this could however mean that activators such as CO can increase in activity thus adding to the complexity of the *tpl/tpr* pleiotropic phenotypes.

It is interesting to note, that an alternatively spliced product for CO exists. This splice variant could produce a truncated protein lacking the middle region and CCT-domain, largely resembling the CO_BB_ artificial microProtein. Due to the presence of a premature termination codon, it is however conceivable that this splice variant might be a target for nonsense-mediated mRNA decay [39]. It can however not be excluded that the splice variant of the *CO* gene, encoding the CO_BB_ microProtein, is expressed under certain environmental conditions. The CO_BB_ microProtein could then feedback-inhibit CO or buffer its activity by sequestering miP1a/b-type proteins. Moreover, it is also possible that miP1a/b-type proteins interact with BBX19 and thus shield CO from engaging in a non-productive complex. Such tripartite switch was recently discovered in the basic helix-loop-helix (bHLH) transcription factor family in which the bHLH protein HBI1 is negatively regulated by the atypical bHLH protein IBH1 which in turn is regulated by the HLH-type microProtein PRE1 [40,41].

The CO_BB_ protein contains both CO-B-Boxes and it can be assumed that these B-Boxes have a high affinity towards each other, explaining why transgenic plants over-expressing this CO protein variant are very late flowering. The same logic would suggest that the strength of interaction of BBX19 with CO is higher than CO with miP1a/b-type proteins. To further explore the use of B-Box proteins and artificial microProteins that are entirely composed of a B-Box, as flowering time regulators, we ectopically expressed COL9 (class II B-Box protein), COL16 (class III B-Box protein) and STO (class IV B-Box protein) including artificial microProteins variants thereof (S9 Fig). None of the recovered transgenic plants exhibited strong flowering time defects, indicating that these proteins cannot trap CO into non-productive complexes. These effects might be attributable to the inability of these proteins to strongly interact with CO. It is interesting to note that BBX19 belongs like STO to the class IV B-Box proteins. Overexpression of STO was recently shown to promote early flowering under both short and long day conditions in a CO-independent manner [42]. These findings suggest that sequences outside the B-Box might contribute to the dominant-negative function of BBX19.

### Evolution of miP1a/b-type microProteins, an example for functional specialization?

Phylogenetic analysis of miP1a/b-type proteins across different genomes revealed that these proteins evolved after the split between monocotyledonous and dicotyledonous lineages. A remarkable difference exists between the regulation of flowering time in rice, a monocotyledonous short-day plant and Arabidopsis, a dicotyledonous long-day plant: The rice CO-orthologue HEADING DATE 1 (Hd1) acts as an activator of flowering time in response to short days (analogous to Arabidopsis CO in long days) but has an additional activity in long days where it acts as a repressor of flowering time [43]. The fact that miP1a/b-type proteins can only be found in dicotyledonous plants implies that they could serve as an example of functional specialization and engage CO into a transcriptional repressor complex.

Analysis of the B-Box domains of miP1a/b-type proteins further revealed that they are structurally different from CO/COL proteins. CO/COL proteins have the following structure [18]: Cx_2_Cx_8_Cx_7_Cx_2_Cx_4_Hx_8_H whereas miP1a/b-type proteins are one residue shorter and have the following structure: Cx_2_Cx_7_Cx_7_Cx_2_Cx_4_Hx_8_H. This latter type of B-Box motif is only shared among five members of the group V BBX-proteins (BBX28, BB29, BBX30 (miP1a), BBX31 (miP1b) and BBX32). Compared to all group V BBX proteins, miP1a/b are much shorter, have a unique amino-terminus and the additional carboxy terminal PFVFL motif. These three features make them remarkably different from all other group V BBX proteins. Furthermore, overexpression of *BBX32* affects light-dependent hypocotyl elongation and not flowering time [44].

### miP1a and miP1b: All different and yet the same?

Both miPa1 and miP1b harbor the first and remnants of a second B-Box domain and contain the carboxyterminal PFVFL motif. They show 65.5% sequence identity towards each other but miP1b is 4 amino acids shorter than miP1a (117aa vs. 121aa). However, all missing residues are found in the sequence after the second B-Box, which might not have biological activity.

The alignment and phylogenetic analysis of the different miP1 orthologues shows a close relationship between the different miP1a and miP1b proteins found in the *Brassicacea* family (S16 Fig). The *Brassicacea* miP1a/b proteins are not only distinguishable by the PFVFL motif from small B-box proteins of other plant families, but also by their overall strong conservation (S14 Fig). Within the *Brassicacea* family we find a clear separation between miP1a and miP1b orthologues and a close analysis of the alignments suggests a common precursor.

Our results show that both miP1a and miP1b act as genuine microProteins and possess the ability to dominantly suppress the activity of CONSTANS. Inhibition of CO results in inability to induce *FLOWERING LOCUS T* in response to long day photoperiods causing these plants to flower extremely late. Furthermore, the late flowering phenotype is for both miP1a and miP1b dependent on the presence of the PFVFL motif. Both *miP1a/b* microProteins are expressed in the vasculature of leaves the place where both CO and FT are active. Mis-expression of miP1b in the vasculature also delays the floral transition, indicating that miP1b is active in this tissue.

Both *miP1a* and *miP1b* microProtein genes exhibit diurnal patterns of expression. In short day conditions both genes peak in expression in the second half of the dark period whereas in long days *miP1b* also peaks around the same time, but *miP1a* expression is high in the first half of the day and then successively decreases (Fig 1G–1K). It is important to note that *miP1b* expression exhibits massive expression changes and the peak of expression is more than 20.000-fold higher compared to the lowest point of expression. It is conceivable that if such large amounts of mRNA are translated into protein, this protein could still be present during the early day thus preventing CO from activating *FT* or together with CO keeping *FT* expression levels low.

Comparative analysis of genes de-regulated in plants ectopically expressing miP1a/b-type microProteins revealed a significant overlap of genes down-regulated in plants ectopically expressing miP1b and plants lacking functional CO protein (Fig 4F). Together with the result that reducing *miP1b* mRNA levels promote the transition to flowering in long days (Fig 2D–2F), these findings suggest that miP1b might be the main microProtein acting on CO activity.

### Conclusion

**Fig 7:**
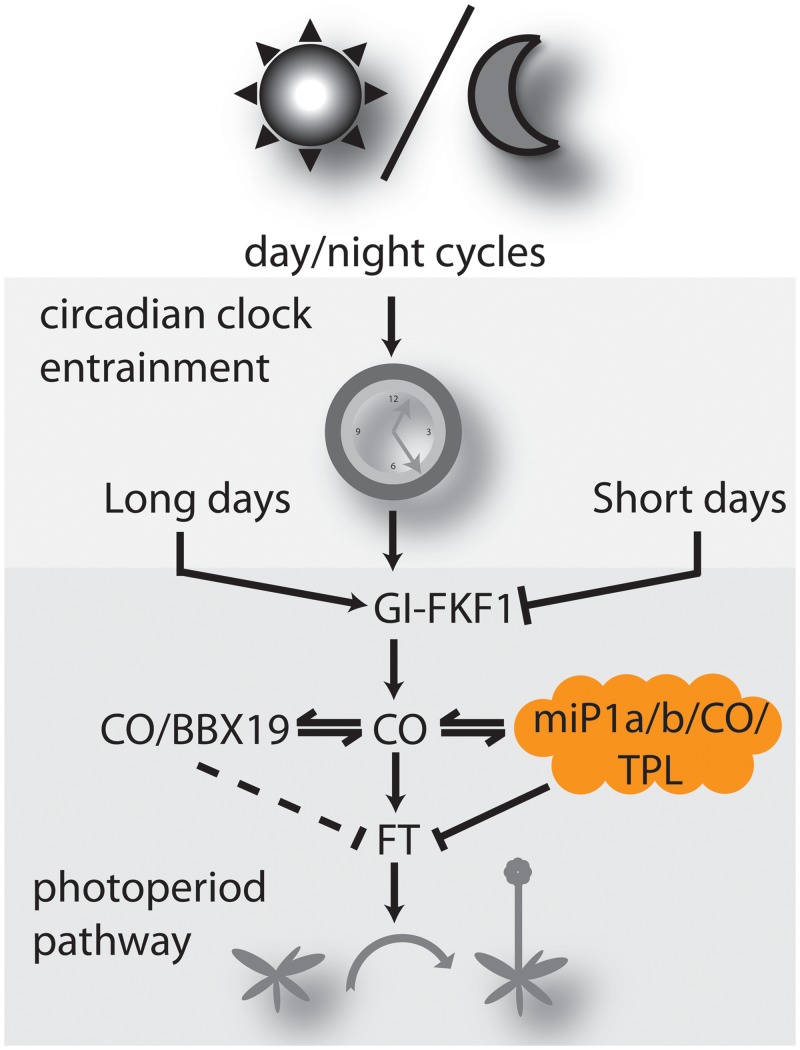
Model depicting the role of microProteins in flowering time regulation. The circadian clock is entrained by day/night cycles. In response to long days, *CO* is activated by GI/FKF1. Increasing levels of CO cause induction of *FT*, which triggers the transition from vegetative to reproductive growth. MiP1a/b act by controlling CO activity. If miP1a/b levels are ectopically high, CO activity is low and flowering is delayed.

MicroProteins are potent regulators of development. Our study identified a number of potential microProteins that affect transcription factors by engaging them in protein complexes that changes their activity. The microProteins that have been identified and characterized to date act by sequestering their targets in non-productive complexes that are either impaired in DNA-binding or hindered from entering the nucleus. Here, we describe two uncharacterized microProteins that act by engaging their targets in complexes with the TOPLESS/TOPLESS-RELATED co-repressor protein, which changes the activity of the transcription factor (Fig 7). Thus, our findings unravel a new role for CONSTANS to engage in a TPL/TPR trimeric complex, which has the potential to fine-tune the flowering response of dicotyledonous plants. The detailed analysis of how TPR/TPL affects flowering is likely complex and requires viable or conditional higher order mutant plants.

## Materials and Methods

### Plant material and growth conditions

To generate transgenic plants overexpressing the cDNAs encoding miP1a/b and the B-Box domains of CO (amino acids 1 to 274) were recombined into the pJAN33 binary vector to create fusion proteins with the FLAG tag. The miP1a* construct was generated by gene synthesis (Life Technologies) with an additional amino terminal myc epitope and flanking Gateway sequences. Transgenic *Arabidopsis thaliana* plants were made using the floral dip method. T1 transgenic plants were isolated after selection with BASTA and lines that contained the T-DNA at a single locus were confirmed by following the segregation ratio in the T2 generation. For flowering-time experiments, seeds were sown on soil, cold-treated for 3 d at 4°C, transferred to a plant growth chamber, and grown in the long day light regime at 20°C. Flowering time was determined by counting the number of rosette leaves at bolting.

### Protein-interaction assays

Interaction of miP1 proteins with the B-Box domains of CO was tested using the Matchmaker Gold yeast-two-hybrid system (Clontech). The coding sequence of either *CO* or the B-Box domains of CO (*CO*_*BB*_) and the coding sequences of *miP1a/b* were recombined into the *pGADT7-GW/pGBKT7-GW* vectors using the LR recombinase mix from Invitrogen. Baits were transformed in the Y2H Gold yeast strain and tested for auto-activation before transformation of the prey plasmids. The screen was performed on SD medium lacking His, Leu, and Trp plus 10 mM 3-aminotriazole.

For the synthesis of the fusion proteins in *E*. *coli* BL21, the coding sequence of *miP1a*, *miP1b* and *ZPR3* were cloned into the *pDEST15* vector, and the coding sequence of *CO* into the *pMALc2* vector. Amylose-Magnetic Beads [NEB E8035S] were used for the purification of MBP-CO. The HRP-conjugated anti-MBP antibody [NEB E8038] was used in a 1:10,000 dilution and the HRP-conjugated GST-antibody [GE Healthcare RPN1236] was used in a 1:5,000 dilution in 5% milk powder-TBS-T. Super signal west pico chemuluminescent substrate [ThermoScietific 34080] was used for the luminescence detection.

For the yeast three hybrid assay the *TOPLESS* coding sequence was recombined into a *pGBKT7-GW* vector and *miP1a* into the *pDRf1-GW* yeast expression vector. The empty vectors or the vectors containing the respective coding sequences were co-transformed into the *pJ69-4α* yeast strain and positive colonies were selected on dropout media without Trp and Ura. The empty *pGADT7-GW* or *pGADT7-GW* with *CO* were transformed into a *YM4271* MATa strain and selected on dropout media without Leu. The presence of the plasmids in the strains was verified by PCR and three positive strains for each transformation were mated for two days at 28°C and then selected on dropout media without Trp, Leu and Ura. Positive colonies were screened on selective media without Trp, Leu, Ura and His with additional 10mM 3-Aminotriazole.

### FRET-FLIM studies

The coding sequence of CONSTANS was recombined into the pK7FWG2 gateway destination vector [45] to express CO with an N-terminal GFP tag. The sequences that code for miP1a, miP1b and miP1a* were recombined into a modified pEarlyGate104 vector (provided by Sabine Müller/Dorothee Stöckle, ZMBP Tübingen) to express N-terminal RFP-fusions in planta. Image and data acquisition was obtained with a Leica TCS SP8, combined with a PicoHarp 300 TCSPC Module and a Sepia Multichannel Picosecond Diode Laser (PDL 808-SC) (Pico-Quant). The samples were excited with a 470 nm pulsed laser (10 MHz) intensity regulated via a Thorlabs Laser Combining Unit (PBH51502/SS/SPL-S6). The emission was recorded from 500 nm to 560 nm in 128 x 128 pixels images with at least 2000 counts/pixel. The fluorescence lifetime measurements were analyzed using the PicoQuant SymphoTime Software (ver. 5.3.2.2). For each nucleus average fluorescence decay profiles were plotted and lifetimes were estimated by fitting the data with a mono-exponential decay function.

### Co-localization experiments

The coding sequences encoding for the CO and TPL proteins were recombined into the previously mentioned vectors for GFP- and RFP-tagged expression from the p35S-promoter. Leaves of N. benthamiana plants were transiently transformed with GFP-CO, RFP-TPL and either pJAN33-miP1a, pJAN33-miP1a* or pJAN33-miP1aΔPFVFL. The localization of GFP and RFP fluorescent proteins in the nuclei of leaf epidermis cells was observed using a Leica SP5-X LCSM. GFP and RFP were excited and the respective emissions were scanned using a sequential scan setting to prevent overlapping fluorescence signals. Intensity measurements were performed with the Leica LAS X software.

### Gene expression studies

For gene expression analysis plants were grown for four weeks in a long day regime at 22°C. RNA was isolated using GeneMATRIX universal RNA purification kit (roboklon, Germany) following manufacturer’s recommendation. Purified RNA (1μg) was used for reverse transcription using ThermoScientific Revert Aid Reverse Transcriptase with oligo-dT primers. Real-time quantitative PCRs were carried out using the ThermoScientific SYBR Green qPCR master mix on a Biorad CFX384. Gene expression levels were calculated using the delta-Ct method and a standard curve relative to GAPDH.

### RNA-Seq transcriptome analysis of Col, *35S*::*FLAG*:*miP1a*, *35S*::*FLAG*:*miP1b* and *co-SAIL* plants

Two samples for each plant type (Col-0, *35S*::*FLAG*:*miP1a*, *35S*::*FLAG*:*miP1b* and *co-SAIL*) were sequenced using Illumina HiSeq2000 and basecalls were performed using HiSeq Control Software v2.0.12.0 (Illumina). For each sample 2.5 to 3.2 Gbases were obtained. Reads were quality checked with RobiNA v1.2.4_build656 and first 10 bases were clipped using Trimmomatic v0.32 [46]. In each sample more than 98% of reads passed the trimming. 63–65% of the surviving reads were successfully mapped to A. thaliana TAIR10 genome sequence and annotation (TAIR) using RobiNA’s Bowtie [47] allowing maximal two mismatches in the seed region. The normalization and statistical evaluation of differential gene expression has been performed using edgeR v2.6.12 [48] with a minimum fold change of 4 and a FDR cut-off of 0.001 and using the Benjamini-Hochberg method [49] for multiple testing correction. The raw data was normalized according to the default procedure and the dispersion was estimated using the auto setting of edgeR. Raw read data and output of statistical analysis were submitted to Gene Expression Omnibus (GSE56811).

### Accession numbers

miP1a/BBX30:At3g21890; miP1b/BBX31:At4g15248; CONSTANS:At5g15840; FLOWERING LOCUS T:At1g65480; COL9:At3g07650; STO:At1g06040; COL16:At1g25440;TPL:At1g15750

## Supporting Information

S1 Data Lists of genes identified by RNA-Seq to be mis-regulated. (XLSX)

S1 Fig Flowchart computational approach. Blue: public available databases; green: resulting Pfam domains; red: microProtein candidates; square: gene identifiers; barrel: Pfam domains.
(PDF)

S2 Fig Number of microProtein candidates with specific Pfam domain domains. TF: transcription factor, ZF: zinc finger, HLH: Helix-loop-helix.
(PDF)

S3 Fig Identification of B-Box-containing microProteins. (A) Phylogenetic tree of all Arabidopsis thaliana B-Box proteins. This minimum evolution tree was generated by aligning B-Box sequences using Muscle 3.2 and 1000 bootstrap replications. (B) Genomic location of Arabidopsis miP1a and miP1b. Both miP1 genes are located close to a COL gene, indicating they evolved by genome-duplication. (C) Domain organization of CONSTANS and CONSTANS-like proteins and the miP1a/b microProteins. (D) Structural models of COL6, CO, miP1a and of CO/miP1a superimposed using MODELLER (E) ClustalW-Alignment of the B-Box domain of COL and miP1a/b proteins.
(PDF)

S4 Fig FRET-FLIM quantification. Average lifetime and standard deviation of GFP-CO co-transformed with different RFP-fusion proteins. The table provides the measured average GFP fluorescence lifetimes, the standard deviation, the significance according to a student’s t-test and the number of nuclei per measurement.
(PDF)

S5 Fig Flowering time of p35S::miP1a* transgenic plants relative to the Col-0 wild type. Average rosettle leaf number of Basta-resistant control plants and 5 independent p35S::miP1a* T1 plants growing under long day conditions (16 light/day).
(PDF)

S6 Fig Flowering time of transgenic plants under short day conditions. Rosette leaf numbers of Col-0, co-sail, ft-10, p35S::miP1a, p35S::miP1b and p35S::COBB plants grown under short day conditions (8 h light / 16 h dark).
(PDF)

S7 Fig Flowering time of long-day grown plants treated with GA. Rosette leaf numbers of Col-0, *co-sail*, *pJAN33*::*miP1a* and *pJAN33*::*miP1b* plants grown under long day conditions (16 h light/ 8 h dark) and either treated with 50 μM GA3 or a control solution containing 0.1% EtOH.
(PDF)

S8 Fig Ectopic expression of *miP1a* in a *co* mutant background does not delay flowering time in long days. Rosette leaf numbers of Col-0, *co-sail*, *pJAN33*::*miP1a* and *co-sail* x *pJAN33*::*miP1a* crosses under long day conditions (16 h light/ 8 h dark).
(PDF)

S9 Fig Flowering of trangenic plants overexpressing different B-Box proteins and artificial B-Box miPs. Average rosette leaf number at the time of bolting in Basta-resistent control plants and T1-plants overexpressing different B-Box proteins or artificial microProteins consisting of their B-Box domains growing under long day conditions.
(PDF)

S10 Fig Characterization of miP1a T-DNA line GABI_KAT_288G08 and transgenic plants over-expressing artificial microRNAs. (A) Rosette leaf number at the time of flower initiation under long (16h light/day; bright green) and short day (8h light/day; dark green) conditions of Col- plants and plants homozygous for the T-DNA insertion GABI_KAT_288G080. (B) Expression of miP1a and miP1b in Col-0 and homozygous T-DNA lines relative to GAPDH determined by qRT-PCR. (C) Rosette leaf number of Col-0 and two independent T2 plant lines expressing a microRNA against miP1a and miP1b grown under long day conditions (16 h light/day).
(PDF)

S11 Fig Physiological responses of Col-0, *35S*::*miP1a* and *35S*::*miP1b* seedlings to salt. Average root length of 7 days old seedlings grown on normal MS media or on MS media containing 50 mM and 100 mM of NaCl.
(PDF)

S12 Fig Flowering time of two independent pSUC2::miP1a transgenic plants relative to Col-0 wild type plants. Average rosette leaf number at the time of flower initiation of Col-0 and T2 plants of two independent transformants growing under long day conditions (16 h light/day).
(PDF)

S13 Fig Comparison of mRNA-seq expression data and qRT-PCR results. Relative expression levels of five differentially regulated genes from the mRNA-seq dataset and a qRT-PCR on cDNA from Col-0, co-sail, p35S::miP1a and p35S::miP1b plants.
(PDF)

S14 Fig Alignment of miP1a/b type proteins from dicot plants. ClustalW-alignment of miP1a/b-type protein sequences.
(PDF)

S15 Fig Phytozome tree. Highlighting the distribution of miP1a/b variants using Phytozome.
(PDF)

S16 Fig Phylogenetic tree. ClustalW-alignment of miP1a/b-type protein sequences. The tree was created using the Neighbor-joining method with 1000 bootstrap replications. Branches equal or bigger 0.25 are shown, branches >0.5 are shown in bold.
(PDF)

S1 Table MicroProteins identified in this study. (DOCX)

S2 Table Sequences of oligonucleotides used in this study. (DOCX)

S1 Text Bioinformatics approach to isolate microProteins in Arabidopsis. (DOCX)
