# Supplementary material for: MicroProtein-Mediated Recruitment of CONSTANS into a TOPLESS Trimeric Complex Represses Flowering in Arabidopsis
Source: PLoS Genet. 2016 Mar 25;12(3):e1005959. doi: 10.1371/journal.pgen.1005959 (PMC4807768; doi:10.1371/journal.pgen.1005959)
Supplement: S1 Fig — Blue: public available databases; green: resulting Pfam domains; red: microProtein candidates; square: gene identifiers; barrel: Pfam domains. (PDF) [file pgen.1005959.s002.pdf]

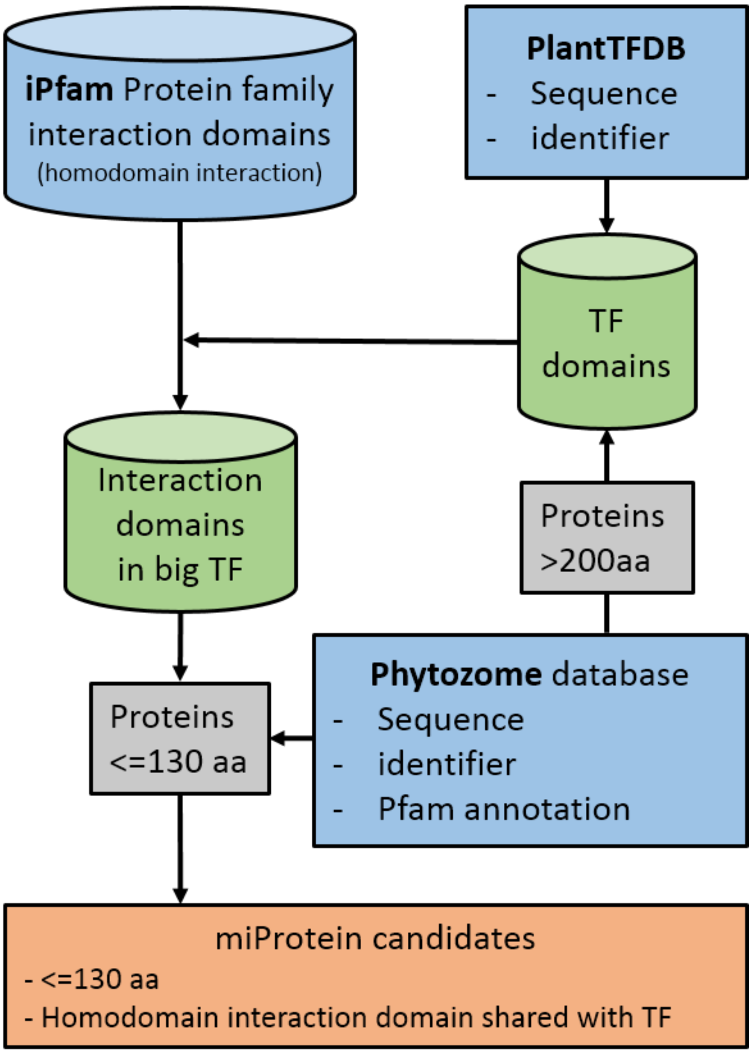

Suppl. Fig. S1: Flowchart computational approach. Blue: public available databases; green: resulting Pfam domains; red: microProtein candidates; square: gene identifiers; barrel: Pfam domains.
