# Supplementary material for: MicroProtein-Mediated Recruitment of CONSTANS into a TOPLESS Trimeric Complex Represses Flowering in Arabidopsis
Source: PLoS Genet. 2016 Mar 25;12(3):e1005959. doi: 10.1371/journal.pgen.1005959 (PMC4807768; doi:10.1371/journal.pgen.1005959)
Supplement: S2 Fig — TF: transcription factor, ZF: zinc finger, HLH: Helix-loop-helix. (PDF) [file pgen.1005959.s003.pdf]

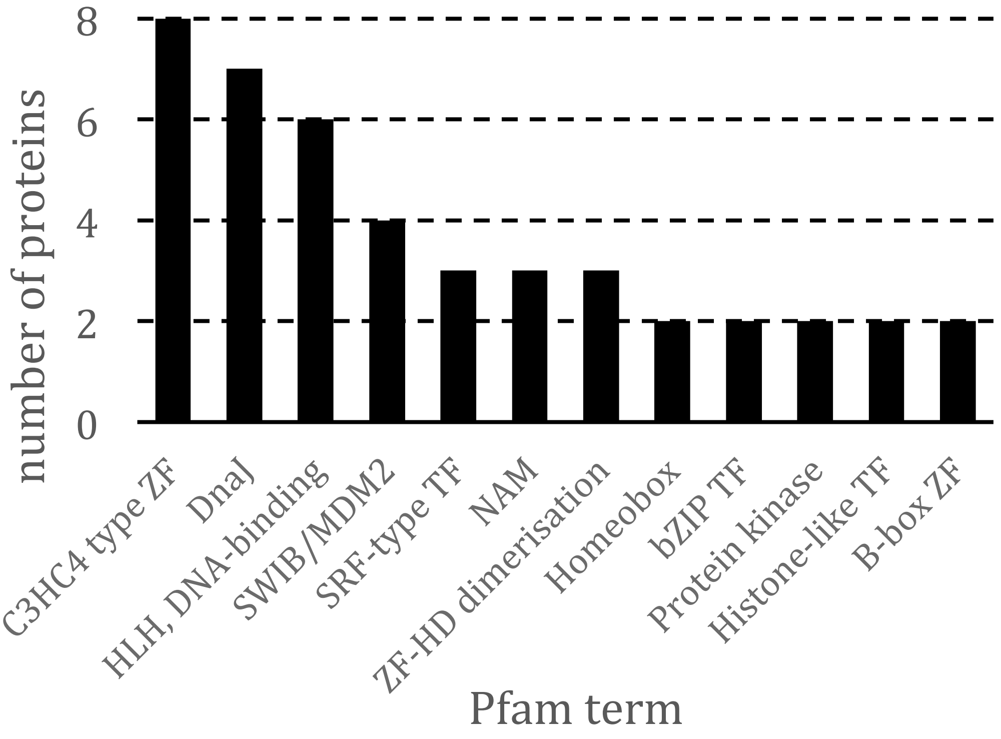

Suppl. Fig. S2: Number of microProtein candidates with specific Pfam domain domains. TF: transcription factor, ZF: zinc finger, HLH: Helix-loop-helix.
