# Supplementary material for: MicroProtein-Mediated Recruitment of CONSTANS into a TOPLESS Trimeric Complex Represses Flowering in Arabidopsis
Source: PLoS Genet. 2016 Mar 25;12(3):e1005959. doi: 10.1371/journal.pgen.1005959 (PMC4807768; doi:10.1371/journal.pgen.1005959)
Supplement: S3 Fig — (A) Phylogenetic tree of all Arabidopsis thaliana B-Box proteins. This minimum evolution tree was generated by aligning B-Box sequences using Muscle 3.2 and 1000 bootstrap replications. (B) Genomic location of Arabidopsis miP1a and miP1b. Both miP1 genes are located close to a COL gene, indicating they evolved by genome-duplication. (C) Domain organization of CONSTANS and CONSTANS-like proteins and the miP1a/b microProteins. (D) Structural models of COL6, CO, miP1a and of CO/miP1a superimposed using MODELLER (E) ClustalW-Alignment of the B-Box domain of COL and miP1a/b proteins. (PDF) [file pgen.1005959.s004.pdf]

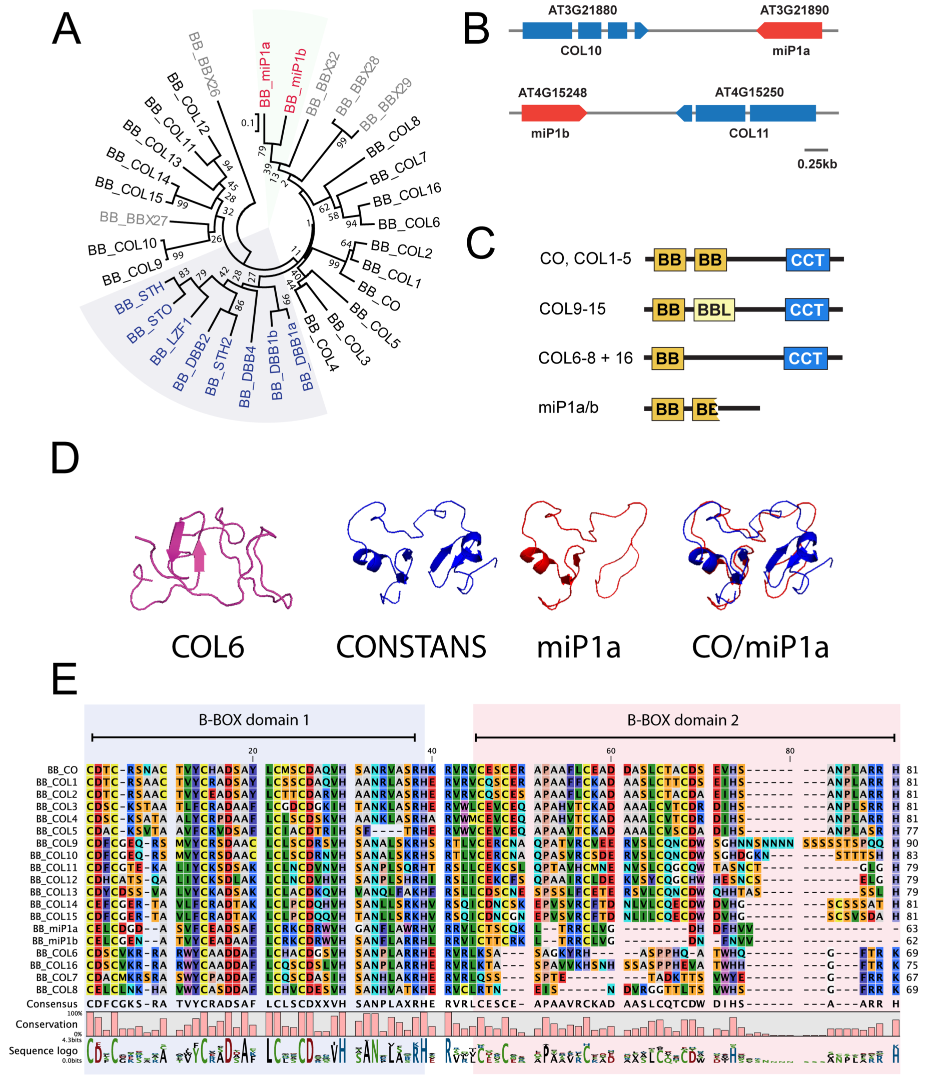

Suppl. Fig. S3 Identification of B-Box-containing microProteins. (A) Phylogenetic tree of all Arabidopsis thaliana B-Box proteins. This minimum evolution tree was generated by aligning B-Box sequences using Muscle 3.2 and 1000 bootstrap replications. **(B)** Genomic location of Arabidopsis miP1a and miP1b. Both miP1 genes are located close to a COL gene, indicating they evolved by genome-duplication. **(C)** Domain organization of CONSTANS and CONSTANS-like proteins and the miP1a/b microProteins. **(D)** Structural models of COL6, CO, miP1a and of CO/miP1a superimposed using MODELLER **(E)** ClustalW-Alignment of the B-Box domain of COL and miP1a/b proteins.
