# Supplementary material for: MicroProtein-Mediated Recruitment of CONSTANS into a TOPLESS Trimeric Complex Represses Flowering in Arabidopsis
Source: PLoS Genet. 2016 Mar 25;12(3):e1005959. doi: 10.1371/journal.pgen.1005959 (PMC4807768; doi:10.1371/journal.pgen.1005959)
Supplement: S4 Fig — Average lifetime and standard deviation of GFP-CO co-transformed with different RFP-fusion proteins. The table provides the measured average GFP fluorescence lifetimes, the standard deviation, the significance according to a student’s t-test and the number of nuclei per measurement. (PDF) [file pgen.1005959.s005.pdf]

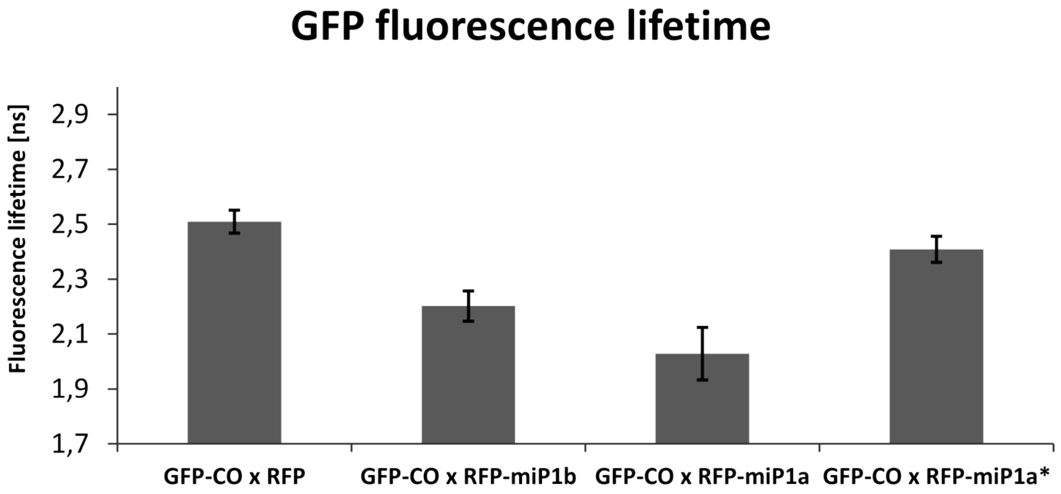

Supp. Table S4

Donor	Acceptor	Lifetime [ns]	s.d.	t-test	Nuclei
GFP-CO	RFP	2,509091	0,04188		11
GFP-CO	RFP-miP1b	2,201667	0,05505	2,67E-12	12
GFP-CO	RFP-miP1a	2,028333	0,09582	1,65E-12	12
GFP-CO	RFP-miP1a*	2,408333	0,04740	4,28E-05	12

Suppl. Fig. S4 FRET-FLIM quantification. Average lifetime and standard deviation of GFP-CO co-transformed with different RFP-fusion proteins. Supplemental table S4 provides the measured average GFP fluorescence lifetime, the standard deviation, the significance according to a student's t-test and the number of nuclei per measurement.
