# Supplementary material for: MicroProtein-Mediated Recruitment of CONSTANS into a TOPLESS Trimeric Complex Represses Flowering in Arabidopsis
Source: PLoS Genet. 2016 Mar 25;12(3):e1005959. doi: 10.1371/journal.pgen.1005959 (PMC4807768; doi:10.1371/journal.pgen.1005959)
Supplement: S5 Fig — Average rosettle leaf number of Basta-resistant control plants and 5 independent p35S::miP1a* T1 plants growing under long day conditions (16 light/day). (PDF) [file pgen.1005959.s006.pdf]

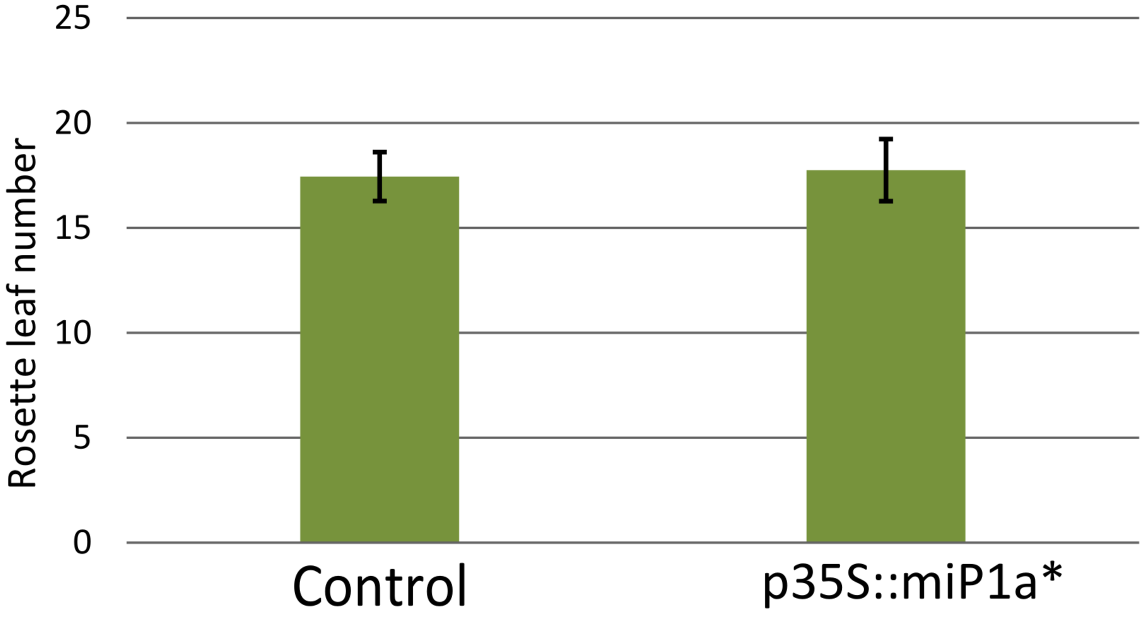

Supp. Fig. S5 Flowering time of p35S::miP1a* transgenic plants relative to the Col-0 wild type. Average rosettle leaf number of Basta-resistant control plants and 5 independent p35S::miP1a* T1 plants growing under long day conditions (16 light/day).
