# Supplementary material for: MicroProtein-Mediated Recruitment of CONSTANS into a TOPLESS Trimeric Complex Represses Flowering in Arabidopsis
Source: PLoS Genet. 2016 Mar 25;12(3):e1005959. doi: 10.1371/journal.pgen.1005959 (PMC4807768; doi:10.1371/journal.pgen.1005959)
Supplement: S6 Fig — Rosette leaf numbers of Col-0, co-sail, ft-10, p35S::miP1a, p35S::miP1b and p35S::COBB plants grown under short day conditions (8 h light / 16 h dark). (PDF) [file pgen.1005959.s007.pdf]

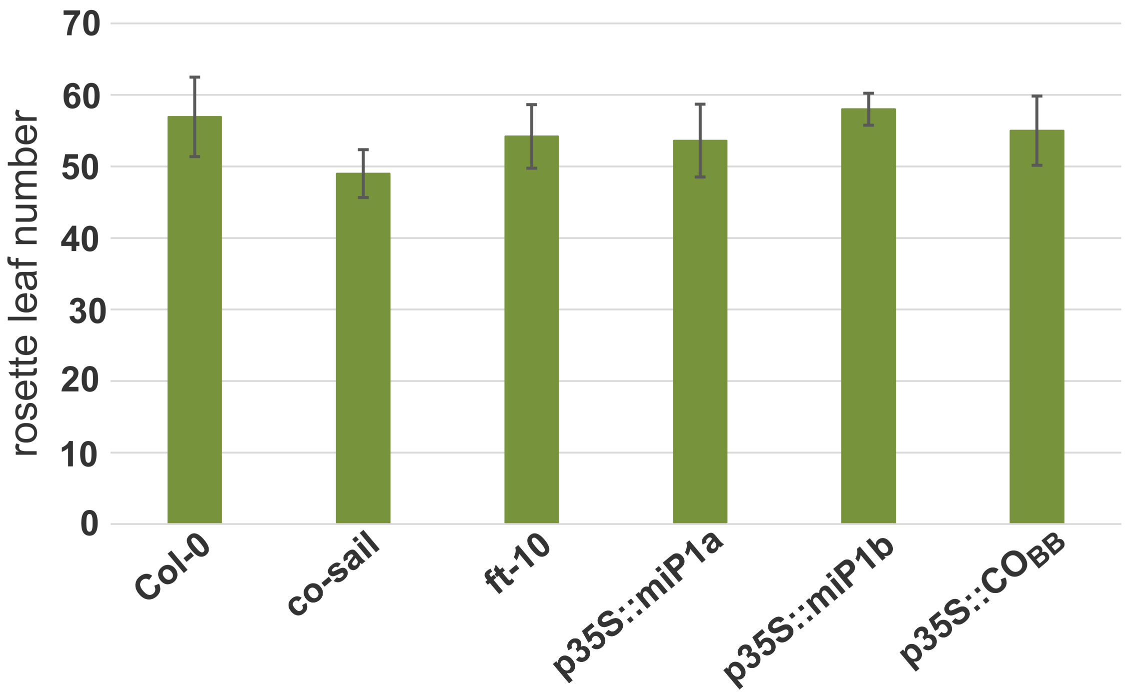

Supp. Fig. S6. Flowering time of transgenic plants under short day conditions. Rosette leaf numbers of Col-0, *co*-sail, *ft*-10, p35S::miP1a, p35S::miP1b and p35S::CO_{BB} plants grown under short day conditions (8 h light / 16 h dark).
