# Supplementary material for: MicroProtein-Mediated Recruitment of CONSTANS into a TOPLESS Trimeric Complex Represses Flowering in Arabidopsis
Source: PLoS Genet. 2016 Mar 25;12(3):e1005959. doi: 10.1371/journal.pgen.1005959 (PMC4807768; doi:10.1371/journal.pgen.1005959)
Supplement: S7 Fig — Rosette leaf numbers of Col-0, co-sail, pJAN33::miP1a and pJAN33::miP1b plants grown under long day conditions (16 h light/ 8 h dark) and either treated with 50 μM GA3 or a control solution containing 0.1% EtOH. (PDF) [file pgen.1005959.s008.pdf]

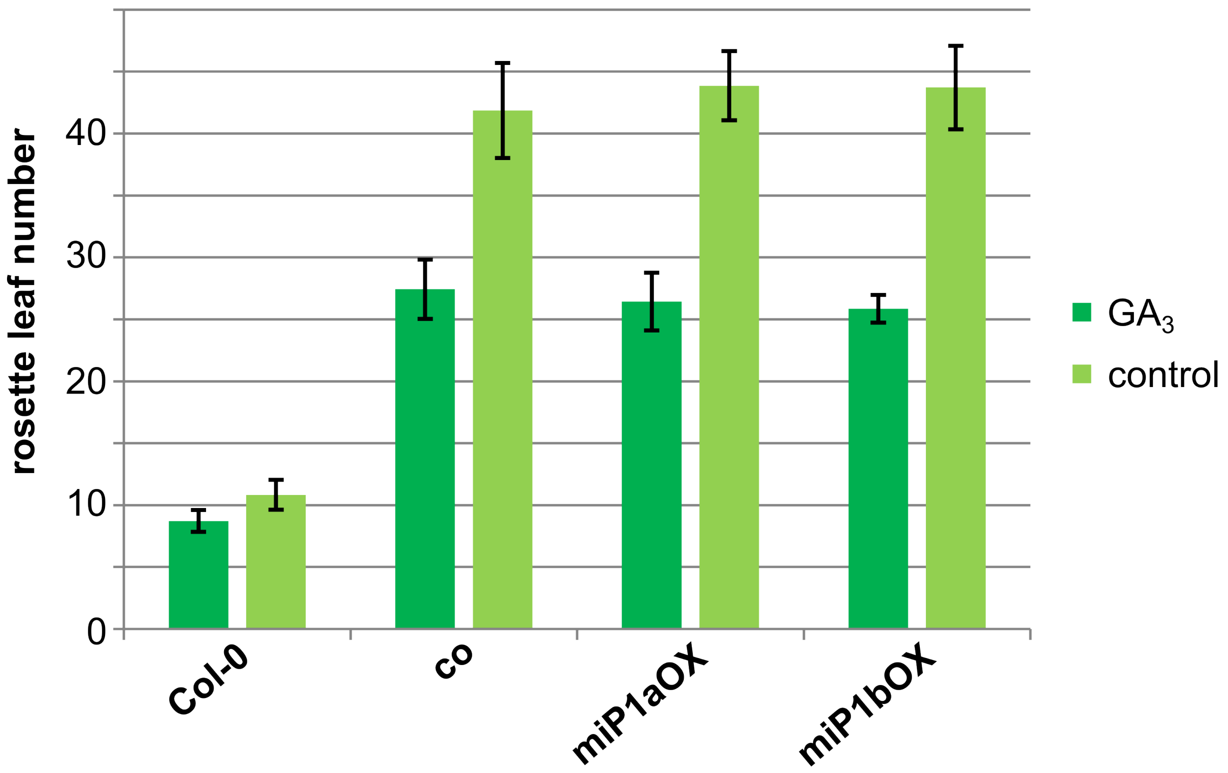

Supp. Fig. S7. Flowering time of long-day grown plants treated with GA.

Rosette leaf numbers of Col-0, *co-sail*, *pJAN33::miP1a* and *pJAN33::miP1b* plants grown under long day conditions (16 h light/ 8 h dark) and either treated with 50 µM GA_3 or a control solution containing 0.1% EtOH
