# Supplementary material for: MicroProtein-Mediated Recruitment of CONSTANS into a TOPLESS Trimeric Complex Represses Flowering in Arabidopsis
Source: PLoS Genet. 2016 Mar 25;12(3):e1005959. doi: 10.1371/journal.pgen.1005959 (PMC4807768; doi:10.1371/journal.pgen.1005959)
Supplement: S8 Fig — Rosette leaf numbers of Col-0, co-sail, pJAN33::miP1a and co-sail x pJAN33::miP1a crosses under long day conditions (16 h light/ 8 h dark). (PDF) [file pgen.1005959.s009.pdf]

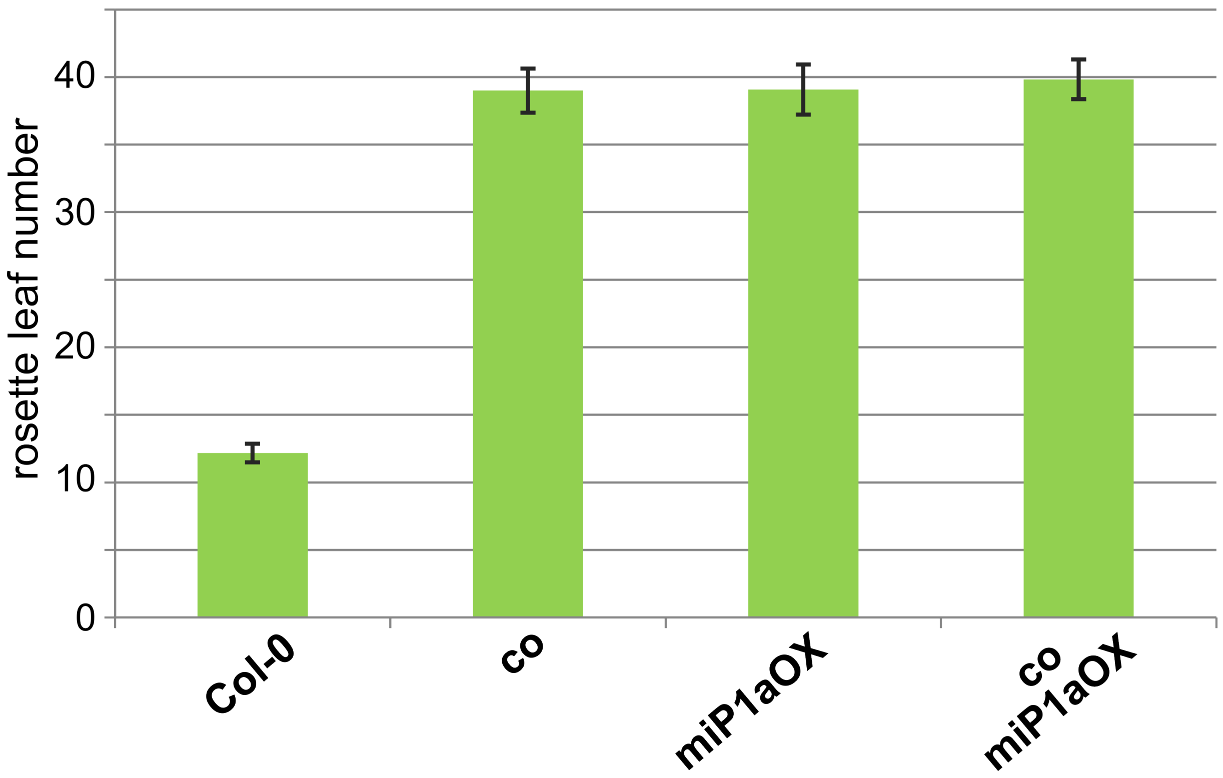

Supp. Fig. S8. Ectopic expression of *miP1a* in a *co* mutant background does not delay flowering time in long days.

Rosette leaf numbers of Col-0, *co-sail*, *pJAN33::miP1a* and *co-sail* x *pJAN33::miP1a* crosses under long day conditions (16 h light/ 8 h dark)
