# Supplementary material for: MicroProtein-Mediated Recruitment of CONSTANS into a TOPLESS Trimeric Complex Represses Flowering in Arabidopsis
Source: PLoS Genet. 2016 Mar 25;12(3):e1005959. doi: 10.1371/journal.pgen.1005959 (PMC4807768; doi:10.1371/journal.pgen.1005959)
Supplement: S9 Fig — Average rosette leaf number at the time of bolting in Basta-resistent control plants and T1-plants overexpressing different B-Box proteins or artificial microProteins consisting of their B-Box domains growing under long day conditions. (PDF) [file pgen.1005959.s010.pdf]

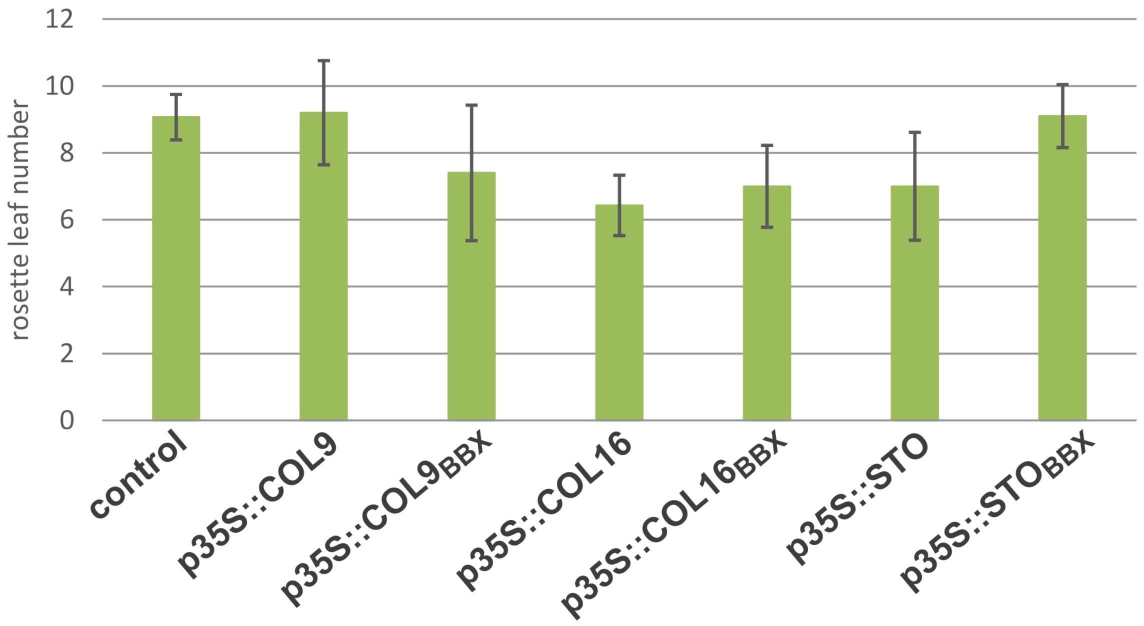


**Supp. Fig. S9. Flowering of trangenic plants overexpressing different B-Box proteins and artificial B-Box miPs.** Average rosette leaf number at the time of bolting in Basta-resistent control plants and  T1-plants overexpressing different B-Box proteins or artificial microProteins consisting of their B-Box domains growing under long day conditions.
