# Supplementary material for: MicroProtein-Mediated Recruitment of CONSTANS into a TOPLESS Trimeric Complex Represses Flowering in Arabidopsis
Source: PLoS Genet. 2016 Mar 25;12(3):e1005959. doi: 10.1371/journal.pgen.1005959 (PMC4807768; doi:10.1371/journal.pgen.1005959)
Supplement: S10 Fig — (A) Rosette leaf number at the time of flower initiation under long (16h light/day; bright green) and short day (8h light/day; dark green) conditions of Col- plants and plants homozygous for the T-DNA insertion GABI_KAT_288G080. (B) Expression of miP1a and miP1b in Col-0 and homozygous T-DNA lines relative to GAPDH determined by qRT-PCR. (C) Rosette leaf number of Col-0 and two independent T2 plant lines expressing a microRNA against miP1a and miP1b grown under long day conditions (16 h light/day). (PDF) [file pgen.1005959.s011.pdf]

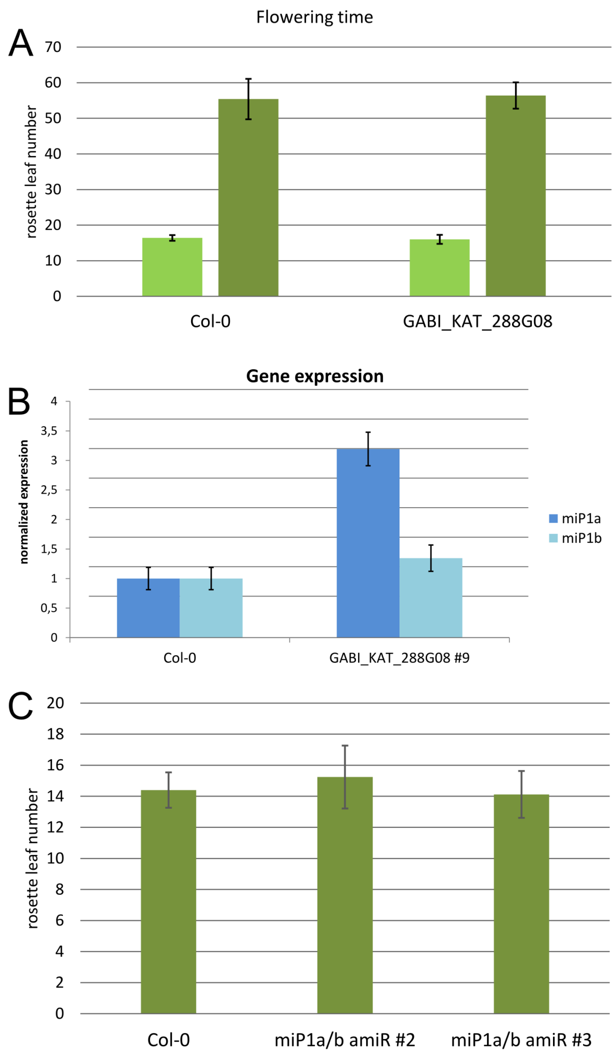

Suppl. Fig. S10 Characterization of miP1a T-DNA line GABI_KAT_288G08 and transgenic plants over-expressing artificial microRNAs. (*A*) Rosette leaf number at the time of flower initiation under long (16h light/day; bright green) and short day (8h light/day; dark green) conditions of Col- plants and plants homozygous for the T-DNA insertion GABI_KAT_288G080. (*B*) Expression of miP1a and miP1b in Col-0 and homozygous T-DNA lines relative to GAPDH determined by qRT-PCR. (*C*) Rosette leaf number of Col-0 and two independent T2 plant lines expressing a microRNA against miP1a and miP1b grown under long day conditions (16 h light/day).
