# Supplementary material for: MicroProtein-Mediated Recruitment of CONSTANS into a TOPLESS Trimeric Complex Represses Flowering in Arabidopsis
Source: PLoS Genet. 2016 Mar 25;12(3):e1005959. doi: 10.1371/journal.pgen.1005959 (PMC4807768; doi:10.1371/journal.pgen.1005959)
Supplement: S11 Fig — Average root length of 7 days old seedlings grown on normal MS media or on MS media containing 50 mM and 100 mM of NaCl. (PDF) [file pgen.1005959.s012.pdf]

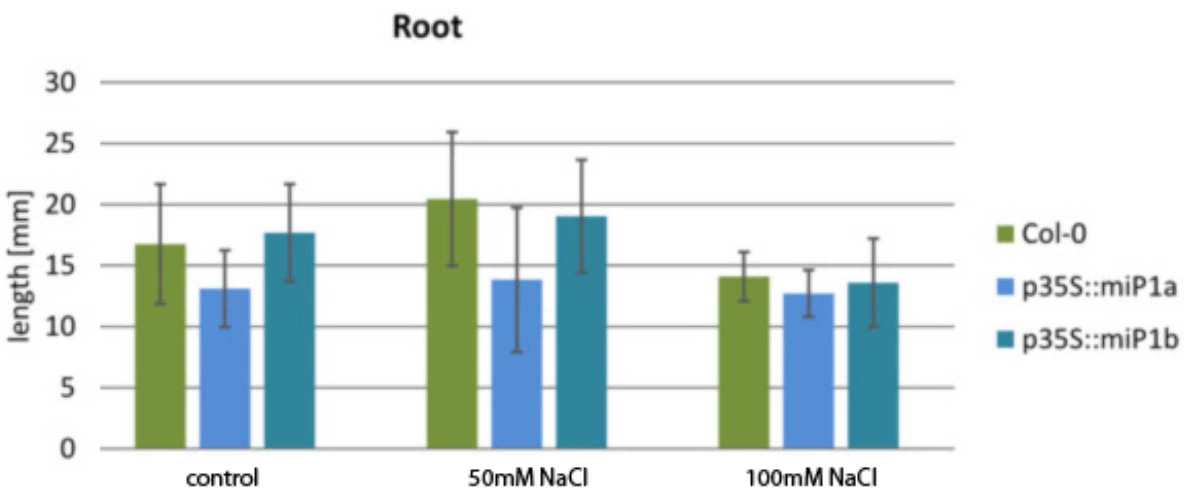

Supp. Fig. S11 physiological responses of Col-0, *35S::miP1a* and *35S::miP1b* seedlings to salt. Average root length of 7 days old seedlings grown on normal MS media or on MS media containing 50 mM and 100 mM of NaCl.
