# Supplementary material for: MicroProtein-Mediated Recruitment of CONSTANS into a TOPLESS Trimeric Complex Represses Flowering in Arabidopsis
Source: PLoS Genet. 2016 Mar 25;12(3):e1005959. doi: 10.1371/journal.pgen.1005959 (PMC4807768; doi:10.1371/journal.pgen.1005959)
Supplement: S12 Fig — Average rosette leaf number at the time of flower initiation of Col-0 and T2 plants of two independent transformants growing under long day conditions (16 h light/day). (PDF) [file pgen.1005959.s013.pdf]

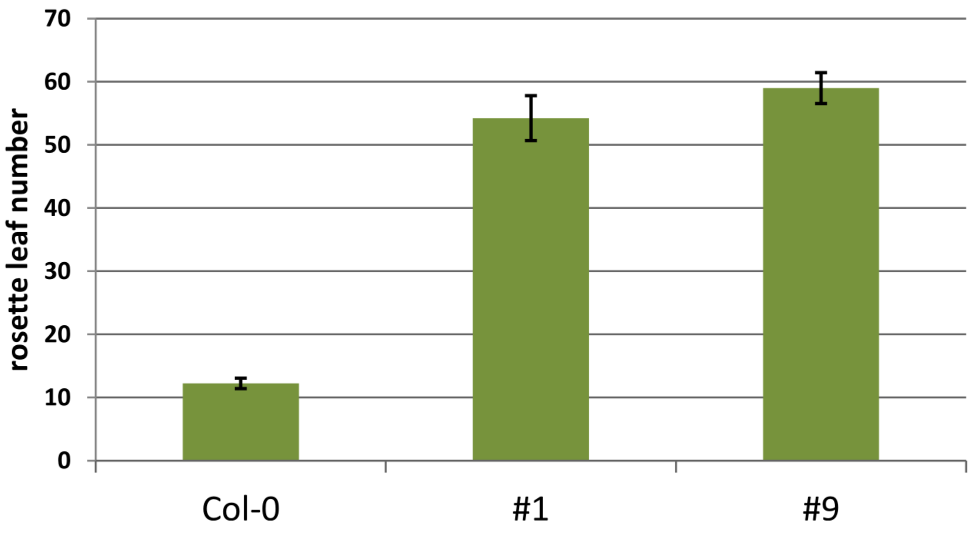


**Supp. Fig. S12. Flowering time of two independent *pSUC2::miP1a* transgenic plants relative to Col-0 wild type plants.** Average rosette leaf number at the time of flower initiation of Col-0 and T2 plants of two independent transformants growing under long day conditions (16 h light/day).
