# Supplementary material for: MicroProtein-Mediated Recruitment of CONSTANS into a TOPLESS Trimeric Complex Represses Flowering in Arabidopsis
Source: PLoS Genet. 2016 Mar 25;12(3):e1005959. doi: 10.1371/journal.pgen.1005959 (PMC4807768; doi:10.1371/journal.pgen.1005959)
Supplement: S13 Fig — Relative expression levels of five differentially regulated genes from the mRNA-seq dataset and a qRT-PCR on cDNA from Col-0, co-sail, p35S::miP1a and p35S::miP1b plants. (PDF) [file pgen.1005959.s014.pdf]

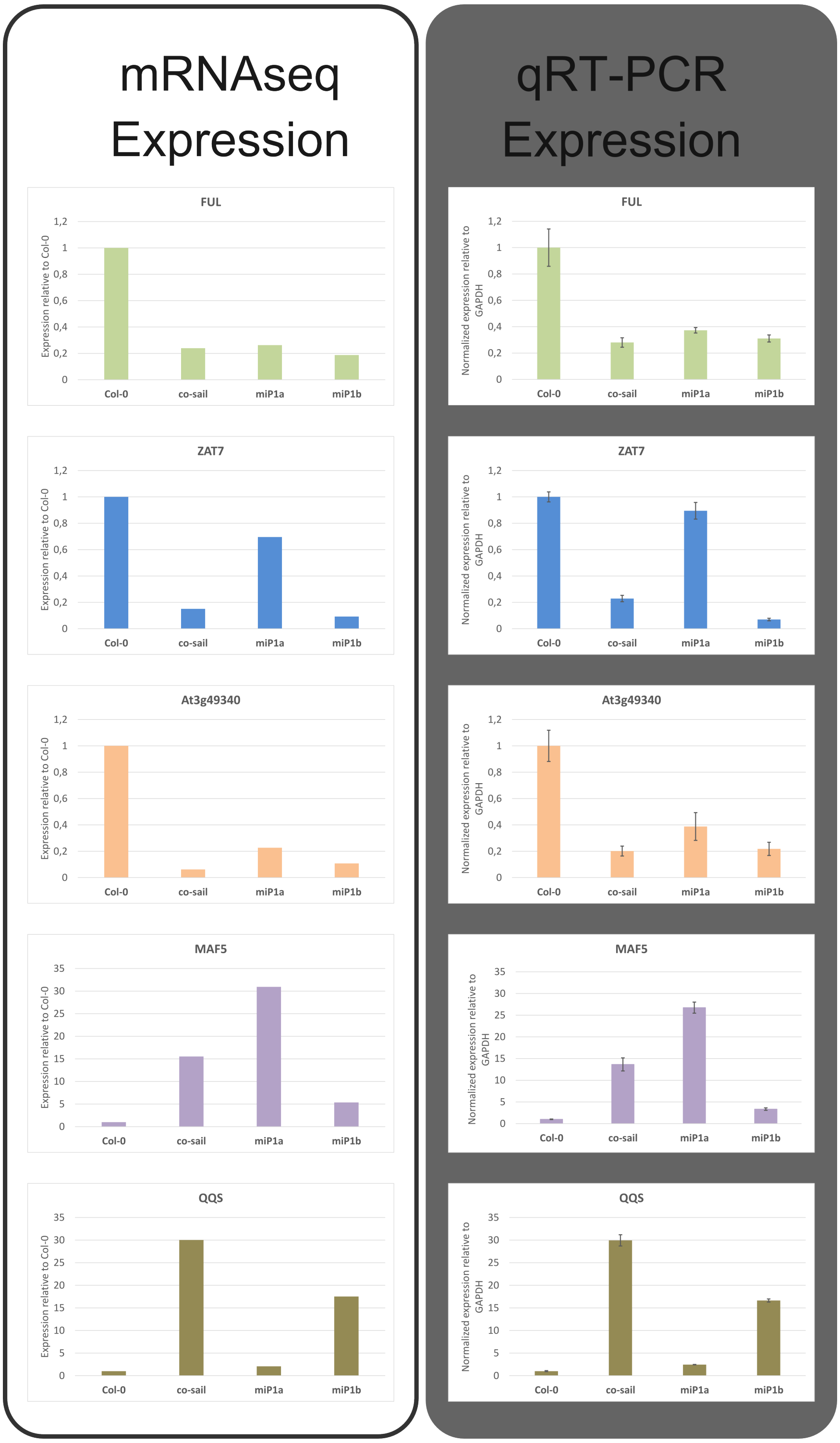


**Supp. Fig. S13. Comparison of mRNA-seq expression data and qRT-PCR results.** Relative expression levels of five differntially regulated genes from the mRNA-seq dataset and a qRT-PCR on cDNA from Col-0, *co*-sail, p35S::miP1a and p35S::miP1b plants.
