# Supplementary material for: MicroProtein-Mediated Recruitment of CONSTANS into a TOPLESS Trimeric Complex Represses Flowering in Arabidopsis
Source: PLoS Genet. 2016 Mar 25;12(3):e1005959. doi: 10.1371/journal.pgen.1005959 (PMC4807768; doi:10.1371/journal.pgen.1005959)
Supplement: S14 Fig — ClustalW-alignment of miP1a/b-type protein sequences. (PDF) [file pgen.1005959.s015.pdf]

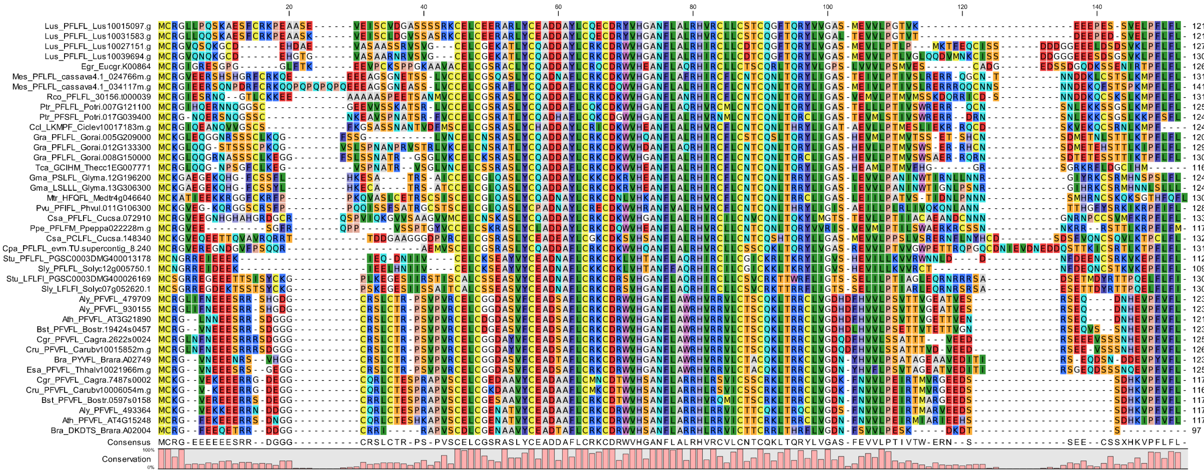

Supp. Fig. S14. Alignment of miP1a/b type proteins from dicot plants.

ClustalW-alignment of miP1a/b-type protein sequences.
