# Supplementary material for: MicroProtein-Mediated Recruitment of CONSTANS into a TOPLESS Trimeric Complex Represses Flowering in Arabidopsis
Source: PLoS Genet. 2016 Mar 25;12(3):e1005959. doi: 10.1371/journal.pgen.1005959 (PMC4807768; doi:10.1371/journal.pgen.1005959)
Supplement: S15 Fig — Highlighting the distribution of miP1a/b variants using Phytozome. (PDF) [file pgen.1005959.s016.pdf]

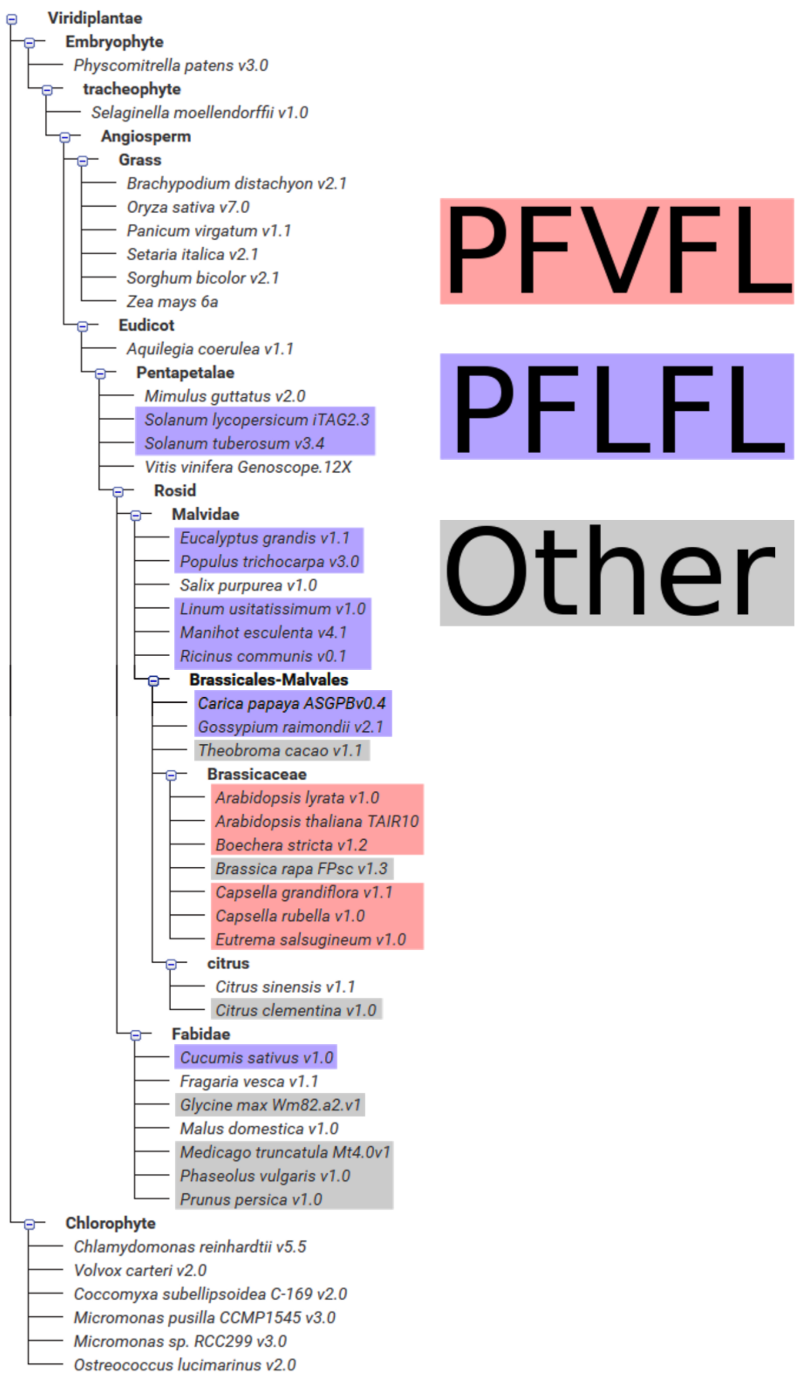

Supplemental Figure S15. Phytozome tree. Highlighting the distribution of miP1a/b variants using Phytozome.
