# Supplementary material for: MicroProtein-Mediated Recruitment of CONSTANS into a TOPLESS Trimeric Complex Represses Flowering in Arabidopsis
Source: PLoS Genet. 2016 Mar 25;12(3):e1005959. doi: 10.1371/journal.pgen.1005959 (PMC4807768; doi:10.1371/journal.pgen.1005959)
Supplement: S16 Fig — ClustalW-alignment of miP1a/b-type protein sequences. The tree was created using the Neighbor-joining method with 1000 bootstrap replications. Branches equal or bigger 0.25 are shown, branches >0.5 are shown in bold. (PDF) [file pgen.1005959.s017.pdf]

last 5 aminoacids sequence logo:

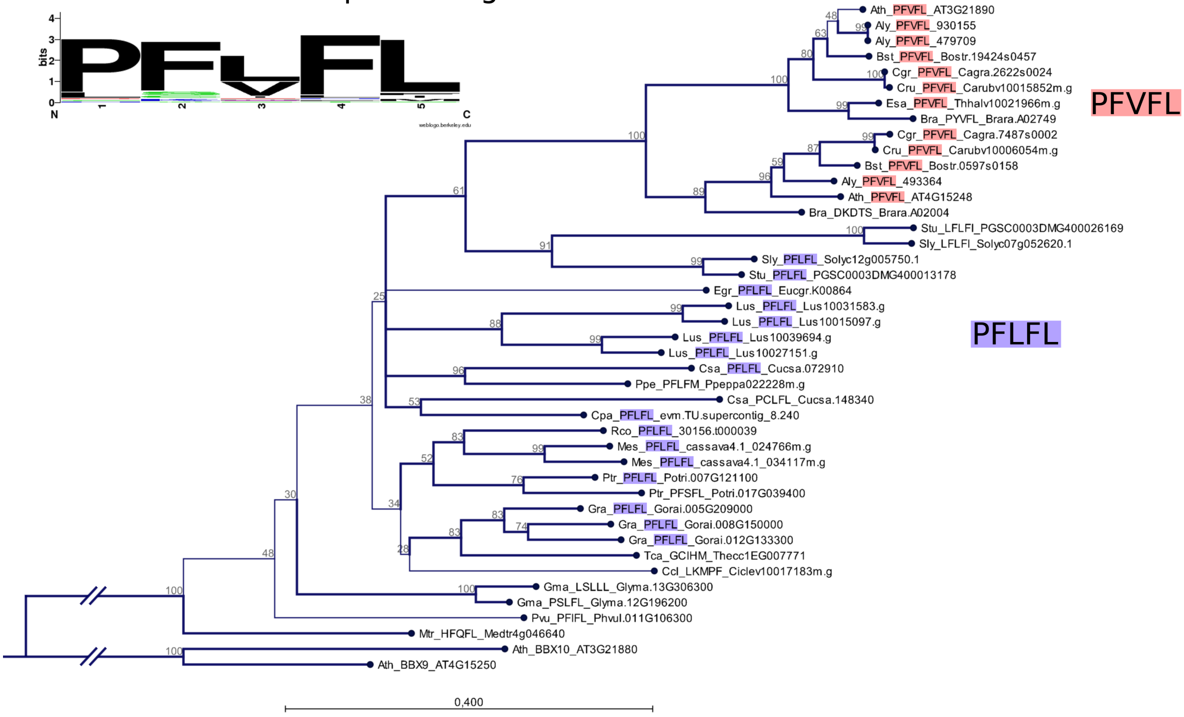

Suppl. Fig. S16. Phylogenetic tree. ClustalW-alignment of miP1a/b-type protein sequences. The tree was created using the Neighbor-joining method with 1000 bootstrap replications. Branches equal or bigger 0.25 are shown, branches >0.5 are shown in bold.
