# Supplementary material for: MicroProtein-Mediated Recruitment of CONSTANS into a TOPLESS Trimeric Complex Represses Flowering in Arabidopsis
Source: PLoS Genet. 2016 Mar 25;12(3):e1005959. doi: 10.1371/journal.pgen.1005959 (PMC4807768; doi:10.1371/journal.pgen.1005959)
Supplement: S1 Table — (DOCX) [file pgen.1005959.s018.docx]

**S1 Table. MicroProteins indentified in this study.**

| **AT-number** | **Annotation** | **Pfam** |
| --- | --- | --- |
| AT1G02210.1 | NAC (No Apical Meristem) domain | PF02365 |
| AT1G18770.1 | RING/U-box superfamily protein | PF00097 |
| AT1G18835.1 | MIF3, mini zinc finger | PF04770 |
| AT1G24580.1 | RING/U-box superfamily protein | PF00097 |
| AT1G26945.1 | KDR, basic helix-loop-helix (bHLH) | PF00010 |
| AT1G31760.1 | SWIB/MDM2 domain superfamily protein | PF02201 |
| AT1G72070.1 | Chaperone DnaJ-domain superfamily protein | PF00226 |
| AT1G74500.1 | ATBS1, BS1, TMO7 | PF00010 |
| AT1G74660.1 | MIF1, mini zinc finger 1 | PF04770 |
| AT1G75390.2 | AtbZIP44, bZIP44, basic leucine-zipper 44 | PF00170 |
| AT2G26320.1 | AGL33, AGAMOUS-like 33 | PF00319 |
| AT2G31215.1 | basic helix-loop-helix (bHLH) | PF00010 |
| AT2G33735.1 | Chaperone DnaJ-domain superfamily protein | PF00226 |
| AT2G35605.1 | SWIB/MDM2 domain superfamily protein | PF02201 |
| AT2G35795.1 | Chaperone DnaJ-domain superfamily protein | PF00226 |
| AT2G38880.4 | ATHAP3, ATNF-YB1, HAP3, HAP3A | PF00808 |
| AT2G38880.6 |  | PF00808 |
| AT3G04410.1 | NAC (No Apical Meristem) domain | PF02365 |
| AT3G09700.1 | Chaperone DnaJ-domain superfamily protein | PF00226 |
| AT3G17609.3 | HYH, HY5-homolog | PF00170 |
| AT3G21890.1 | B-box type zinc finger family protein | PF00643 |
| AT3G28917.1 | MIF2, mini zinc finger 2 | PF04770 |
| AT3G47710.1 | BHLH161, BNQ3, BANQUO 3 | PF00010 |
| AT3G51325.1 | RING/U-box superfamily protein | PF00097 |
| AT3G56770.2 | basic helix-loop-helix (bHLH) | PF00010 |
| AT3G62190.2 | Chaperone DnaJ-domain superfamily protein | PF00226 |
| AT4G00305.1 | RING/U-box superfamily protein | PF00097 |
| AT4G04632.1 | Protein kinase superfamily protein | PF00069 |
| AT4G12190.1 | RING/U-box superfamily protein | PF00097 |
| AT4G15248.1 | B-box type zinc finger family protein | PF00643 |
| AT4G24204.1 | RING/U-box superfamily protein | PF00097 |
| AT4G26810.1 | SWIB/MDM2 domain superfamily protein | PF02201 |
| AT4G26810.2 |  | PF02201 |
| AT5G01070.1 | RING/FYVE/PHD zinc finger superfamily protein | PF00097 |
| AT5G03030.1 | Chaperone DnaJ-domain superfamily protein | PF00226 |
| AT5G05770.1 | WOX7, WUSCHEL related homeobox 7 | PF00046 |
| AT5G15160.1 | BHLH134, BNQ2, BANQUO 2 | PF00010 |
| AT5G16650.1 | Chaperone DnaJ-domain superfamily protein | PF00226 |
| AT5G18037.1 | NAC (No Apical Meristem) domain | PF02365 |
| AT5G27050.1 | AGL101, AGAMOUS-like 101 | PF00319 |
| AT5G27810.1 | MADS-box transcription factor family protein | PF00319 |
| AT5G41440.1 | RING/U-box superfamily protein | PF00097 |
| AT5G46010.1 | Homeodomain-like superfamily protein | PF00046 |
| AT5G57565.2 | Protein kinase superfamily protein | PF00069 |
