# Supplementary material for: MicroProtein-Mediated Recruitment of CONSTANS into a TOPLESS Trimeric Complex Represses Flowering in Arabidopsis
Source: PLoS Genet. 2016 Mar 25;12(3):e1005959. doi: 10.1371/journal.pgen.1005959 (PMC4807768; doi:10.1371/journal.pgen.1005959)
Supplement: S2 Table — (DOCX) [file pgen.1005959.s019.docx]

**S2 Table. Sequences of oligonucleotides used in this study.**

| Name | Sequence | AGI code |
| --- | --- | --- |
| COF | ggggacaagtttgtacaaaaaagcaggctgcATGTTGAAACAAGAGAGTAACG | At5g15840.1 |
| COR | ggggaccactttgtacaagaaagctgggtcGAATGAAGGAACAATCCCAT | At5g15840.1 |
| COSBBr | ggggaccactttgtacaagaaagctgggtcTCACCCTGCTGCGTTATGGG | At5g15840.2 |
| miP1b F | ggggacaagtttgtacaaaaaagcaggctgcATGTGTAGAGGGTTTGAGAA | At4g15248.1 |
| miP1b R | ggggaccactttgtacaagaaagctgggtcTCAGAGAAACACAAAGGGAA | At4g15248.1 |
| miP1a F | ggggacaagtttgtacaaaaaagcaggctgcATGTGTAGAGGCTTGAATAA | At3g21890.1 |
| miP1a R | ggggaccactttgtacaagaaagctgggtcTCAGAGAAAAACAAACGGAAC | At3g21890.1 |
| miP1a R -PFVFL | TCAAACCTCATGATTATCTTGTT |  |
| miP1b R -PFVFL | TCACATAGTAGTGATCACAAAATT |  |
| pmiP1af | ggggacaagtttgtacaaaaaagcaggctgctgtagagaaatgtcgtgggtttt |  |
| pmiP1ar | ggggaccactttgtacaagaaagctgggtctgaggaaagaagatttgggaat |  |
| pmiP1bf | ggggacaagtttgtacaaaaaagcaggctgcgaacctataaagaatatttctcgaatg |  |
| pmiP1br | ggggaccactttgtacaagaaagctgggtctctttctttgtctctcttgtgttca |  |
| TPLf | CACCATGTCTTCTCTTAGTAGA | At1g15750.1 |
| TPLr | TCTCTGAGGCTGATCAGATGCA |  |
| STO F | ggggacaagtttgtacaaaaaagcaggctgcATGAAGATACAGTGTGATGT | At1g06040.1 |
| STO_BBX R | ggggaccactttgtacaagaaagctgggtcTTACATATAGTTGAGGTCAGAGC |  |
| COL9 F | ggggacaagtttgtacaaaaaagcaggctgcATGGGTTACATGTGTGACTT | At3g07650.1 |
| COL9_BBX R | ggggaccactttgtacaagaaagctgggtcTCAAATGGAAGCGAGTTCTGAG |  |
| COL16 F | ggggacaagtttgtacaaaaaagcaggctgcATGATGAAAAGTTTGGCGAA | At1g25440.1 |
| COL16_BBX R | ggggaccactttgtacaagaaagctgggtcTCAGTGGTTGCTATGCTTTA |  |
| miR173ts BP F | ggggacaagtttgtacaaaaaagcaggctgcGTGATTTTTCTCTACAAGCGAA |  |
| MIGS miP1a 1F | GTGATTTTTCTCTACAAGCGAAATGTGTAGAGGCTTGAATAA |  |
| MIGS miP1b 1F | GTGATTTTTCTCTACAAGCGAAATGTGTAGAGGGTTTGAGAA |  |
| MIGS C-miP1a/b 1F | GTGATTTTTCTCTACAAGCGAAAATTTTCTAGCTNGGAGACA |  |
| **qPCR** |  |  |
| qFTf | CAGGAATTCATCGTGTCGTG | At1g65480.1 |
| qFTr | AGCCACTCTCCCTCTGACAA | At1g65480.1 |
| qCO.1/2 1F | AAACTGCAGCGTACCACAGA | At5g15840.1/2 |
| qCO.1 1R | GGATGAAATGTATGCGTTATGG | At5g15840.1 |
| qCO.2 1R | CTGCTGCGTTATGGGAAGAT | At5g15840.2 |
| qmiP1a 1F | GCAGAAGAAGTGACGGAGGA | At3g21890.1 |
| qmiP1a R | CGCGTGAGTTTCTGACAAGA | At3g21890.1 |
| qmiP1a* 1R | GAGTTTCTGGGCAGAAGTGG |  |
| qmiP1b 1R | TGCTATCATCCTTATCTCCGGT | At4g15248.1 |
| qmiP1b R | ACGAGTTAGCTTCCGACAGG | At4g15248.1 |
| FULf | GGTCATTTCAGGGTTGTCGT | At5g60910.1 |
| FULr | CGAAGAGTTTGCCTTTGGAA | At5g60910.1 |
| ZAT7f | GGGAGATGAACGTGTTTTCC | At3g46090.1 |
| ZAT7r | TCTCCTCATGTGACCACCAA | At3g46090.1 |
| At3g49340f | CGAGGAATTTAAGGCGAGGT | At3g49340.1 |
| At3g49340r | ATCTTCGTCATGCCTTCCAC | At3g49340.1 |
| At2g26400f | TTTGGACAAACTTGCAGAGC | At2g26400.1 |
| At2g26400r | CGAGGCAGTAACGGATCTCT | At2g26400.1 |
| MAF5f | TTCAGGATCTCCGACCAGTT | At5g65080.1/2 |
| MAF5r | GACGGAGGATCCACAGAGAA | At5g65080.1/2 |
| QQSf | TTTCGATCTGTCAGCCATTG | At3g30720.1 |
| QQSr | CTGGTCGCTGTGGAGAAAAT | At3g30720.1 |
| qmiP1a 5'UTR F | TTTCCTCAATATCACCCAGAAGA | At3g21890.1 |
| qmiP1b 5'UTR F | GAACACAAGAGAGACAAAGAAAGAG | At4g15248.1 |
