# Supplementary material for: MicroProtein-Mediated Recruitment of CONSTANS into a TOPLESS Trimeric Complex Represses Flowering in Arabidopsis
Source: PLoS Genet. 2016 Mar 25;12(3):e1005959. doi: 10.1371/journal.pgen.1005959 (PMC4807768; doi:10.1371/journal.pgen.1005959)
Supplement: S1 Text — (DOCX) [file pgen.1005959.s020.docx]

**Supplementary text S1 Text.**

**Bioinformatics approach to isolate microProteins in Arabidopsis**

In order to identify novel microProtein regulators of transcription factors in the model plant *Arabidopsis thaliana*, we chose a computational approach (Suppl. Figure 1). Pfam domains of all 2297 transcription factors in the plantTFDB v3.0 ([Jin et al. 2014](#_ENREF_3)) were fetched from the annotation file of the Phytozome database v9.1 ([Swarbreck et al. 2008](#_ENREF_4)). To exclude small targets, transcription factors smaller than 200 amino acids were ignored, remaining 1985 transcription factors. The resulting set of 257 Pfam domains was filtered for interchain homodomain interaction domains present in the iPfam database v1.0 ([Finn et al. 2014](#_ENREF_2)). Domain interactions in the iPfam database are calculated using all know structures in the Protein Data Bank ([Berman et al. 2003](#_ENREF_1)). For the dimerization region of zinc finger homeodomain plant transcription factors (PF04770) no PDB structures are available, but we added the domain to the list of interaction domains. Following, proteins with the 42 homodomain interaction domains of transcription factors in the Phytozome database v9.1 were selected. But since all known microProteins are small, we limited the size of the investigated proteins to be shorter than 130 amino acids. We were able to identify 44 proteins derived from 42 genes (Suppl. Table 1) with 12 different Pfam interaction domains (Suppl. Figure 2). Due to our limitation of protein size and usage of imperfect databases (plantTFDB, iPfam and Phytozome) we assume that our analysis is not complete and might lack other potential microProteins.

Magnani et al. (2014) recently produced a list of truncated transcription factor-like proteins using a bioinformatics approach. They focused on proteins that share sequence similarity with transcription factors (TFs) but lack a known DNA-binding domain and are not larger or bear more domains than their putative target TFs. They found more than 400 proteins in *A. thaliana* fulfilling these criteria. The main differences of our approach to Magnani et al. (2014) are the presence of a protein-protein-interaction domain (PPID) combined with a very short protein size (max. length 130 vs 500 amino acids). The microProteins identified in this approach have only a single domain, the PPID. Additionally our search is more restrictive and conservative and we chose to use already annotated proteins. Therefore, this screen enriches for small proteins that have the potential to act in a dominant-negative fashion.

**Supplementary references**

Benjamini, Y., and Hochberg, Y. 1995. Controlling the false discovery rate: a practical and powerful approach to multiple testing. Journal of the Royal Statistical Society Series B, 57: 289–300.

Berman H, Henrick K, Nakamura H. 2003. Announcing the worldwide Protein Data Bank. Nature structural biology 10: 980.

Finn RD, Miller BL, Clements J, Bateman A. 2014. iPfam: a database of protein family and domain interactions found in the Protein Data Bank. Nucleic Acids Res 42: D364-373.

Jin J, Zhang H, Kong L, Gao G, Luo J. 2014. PlantTFDB 3.0: a portal for the functional and evolutionary study of plant transcription factors. Nucleic Acids Res 42: D1182-1187.

Langmead, B., et al. 2009.Ultrafast and memory-efficient alignment of short DNA sequences to the human genome. Genome Biol 10 (3): p. R25

Lohse M, Bolger AM, Nagel A, Fernie AR, Lunn JE, Stitt M, Usadel B. 2012. RobiNA: A user-friendly, integrated software solution for RNA-Seq-based transcriptomics. Nucleic Acids Res. 40 (Web Server issue):W622-7.

Magnani E, de Klein N, Nam HI, Kim JG, Pham K, Fiume E, Mudgett MB, Rhee SY. 2014. A comprehensive analysis of microProteins reveals their potentially widespread mechanism of transcriptional regulation. Plant Physiol. 2014 Mar 10.

Robinson MD, McCarthy DJ, Smyth GK. 2010. edgeR: a Bioconductor package for differential expression analysis of digital gene expression data. Bioinformatics 26: 139-14.

Swarbreck D, Wilks C, Lamesch P, Berardini TZ, Garcia-Hernandez M, Foerster H, Li D, Meyer T, Muller R, Ploetz L et al. 2008. The Arabidopsis Information Resource (TAIR): gene structure and function annotation. Nucleic Acids Res 36: D1009-1014.
